# Evaluation of the quality, reliability, and readability of ChatGPT-4 responses related to the treatment and rehabilitation of children with cerebral palsy

**DOI:** 10.1007/s00431-026-06979-3

**Published:** 2026-04-24

**Authors:** Rabia Zorlular, Ali Zorlular

**Affiliations:** Department of Physiotherapy and Rehabilitation, Faculty of Health Sciences, Nigde Omer Halisdemir University, Nigde, Turkey

**Keywords:** Artificial intelligence, ChatGPT, Cerebral palsy, Exercise, Rehabilitation

## Abstract

This study aims to evaluate the reliability, quality, and readability of ChatGPT-4’s responses to questions about cerebral palsy (CP) and rehabilitation strategies. The 56 most frequently asked questions by families about CP, its treatment, and rehabilitation strategies were divided into five categories (A1-5) and asked in ChatGPT-4. The reliability, quality, and readability of the responses were assessed by two researchers, respectively, using the modified DISCERN (mDISCERN) tool, the Global Quality Scale (GQS), and the Flesch Reading Ease Scale (FRE). Median (IQR) values for mDISCERN ranged from 3 (3–3) in A5 to 4 (3–4) in A1, while GQS values ranged from 3 (3–3.5) in A2 to 3.5 (3–4) in A1. The mean readability values assessed with FRE ranged from 31.47 ± 12.92 to 44.84 ± 17.39. No statistically significant differences were observed between the categories. The ICC values suggested very good agreement for both scales, with 0.851 for the mDISCERN total score and 0.824 for the GQS total score. *Conclusion*: This study suggests that ChatGPT-4 provides moderate reliability and generally acceptable quality of responses regarding CP treatment and rehabilitation. FRE scores indicated that many responses were difficult for families to understand. While ChatGPT-4 may serve as a supportive source of general information, its outputs should be interpreted with caution, particularly in clinical contexts, and supervision and oversight by healthcare professionals remain essential for safe and effective use.

**Table:** 

**What's Known:** • *Children with cerebral palsy (CP) receive lifelong rehabilitation. Exercise plays a fundamental role in maintaining functional independence and quality of life, as well as physical development. The use of AI-based tools such as ChatGPT-4 for health information is increasingly widespread; however, uncertainties regarding the reliability, quality,and readability of these tools remain.* **What is New:** • *Our study shows that ChatGPT-4’'s responses to questions about treatment and rehabilitation strategies for children with CP have moderate to good reliability and quality, but low readability.*

**Supplementary Information:**

The online version contains supplementary material available at 10.1007/s00431-026-06979-3.

## Introduction

Cerebral palsy (CP) is a neurodevelopmental disorder caused by damage to the developing brain. While the damage is not progressive, the resulting secondary impairments (muscle function, motor skills) can be progressive [1]. The prevalence of CP is estimated to range from 1.4 to 1.8 per 1000 live births in developed countries [2], whereas in underdeveloped and developing countries, the prevalence is higher, ranging from 2.95 to 3.4 per 1000 live births [3]. It is a heterogeneous umbrella term affecting not only motor functions but also cognitive skills, behavior, hearing and vision abilities, and academic performance [4]. Individuals with CP experience lifelong effects that influence their ability to perform daily self-care tasks, engage in play, and participate in educational, social, and community activities [5].

While CP cannot be cured definitively, its long-term effects can be mitigated with ongoing physiotherapy, family education, medical interventions, and therapeutic support. The primary goal of rehabilitation programs in children with CP is to increase functional abilities and promote optimal developmental progress [6]. In accordance with World Health Organization guidance, maintaining and improving the range of motion is considered a fundamental goal in the rehabilitation process [7]. Parents, as the primary caregivers of children with CP, must cope with numerous stressors throughout the long course of treatment and are often engaged in an ongoing search for the most effective therapeutic options. Children with CP necessitate more health interventions than their healthy peers and experience more difficulties due to secondary problems caused by the disease [8]. In addition to caring for children with CP daily, parents must also manage their children’s complex health needs and rehabilitation processes, which require significant time, effort, and regular follow-up. Parents often seek information about health issues and rehabilitation from physical therapists, doctors, and other healthcare professionals; however, due to limitations in access, time, or available resources, not all families may receive timely answers.

The rapid evolution of artificial intelligence (AI) has enabled the rise of large language model (LLM)–based chatbots, which create natural, human-like responses and address queries spanning multiple fields, including healthcare. Among these, ChatGPT — one of the most widely used models — is a language model developed by OpenAI that utilizes deep learning techniques to produce human-like responses to natural language input [9]. Its ability to generate and understand structurally related and coherent responses has led to its discovery and frequent use in various sectors, particularly healthcare [9]. Particularly, AI-based chatbots are increasingly being used to support clinical functions such as screening, diagnosis, and treatment. Meanwhile, researchers suggest that the healthcare industry’s AI-based approach could reduce its growing carbon footprint [10]. The use of AI can reduce the carbon footprint of the healthcare system by reducing the need for patients to go to hospitals for minor problems and ambiguities. However, many academics and healthcare professionals emphasize that chatbots cannot technically diagnose medical problems, recommend treatments, or replace professional opinions [11, 12], as LLMs have inherent limitations, including the production of false or fabricated information, commonly referred to as hallucinations.

AI-based tools such as ChatGPT have the potential to support both families of children with CP and rehabilitation professionals. For families, these tools may provide rapid access to general information about rehabilitation strategies, exercise programs, and treatment options, particularly when direct access to healthcare professionals is limited. For rehabilitation professionals, AI systems may serve as supportive resources for patient education by addressing frequently asked questions and reinforcing information provided during clinical consultations. Therefore, evaluating the reliability, quality, and readability of AI-generated responses to common questions related to the treatment and rehabilitation of children with CP is important. This study aims to evaluate the ability of ChatGPT-4 to answer questions related to the treatment and rehabilitation of children with CP. Specifically, the quality, reliability, and readability of the information generated by ChatGPT-4 were assessed in response to questions commonly asked by families of children with CP.

## Methods

No ethical approval was required, since no human participants or identifiable data were involved. AI was used only to generate responses to the study questions as part of the research design. AI tools were not used in the writing, analysis, or preparation of the manuscript, and all sections of the manuscript were prepared by the authors.

### Question design, categorization, and processing

Between September 1 and 15, 2025, two researchers with PhD degrees in Physiotherapy and Rehabilitation compiled frequently asked questions encountered during rehabilitation sessions with children with CP and their families. Google Trends was used as a supplementary tool to identify commonly searched topics. This process generated a pool of 90 questions related to general information about CP, exercise and rehabilitation strategies, medical and orthopedic interventions, gait and balance, and hand function. After the initial question pool was created, the questions were screened according to predefined inclusion and exclusion criteria. Questions that were clinically irrelevant, unclear, or similar in content were excluded, and duplicate items were removed. The remaining questions were reviewed by both researchers, and the final set of 56 questions was determined through consensus, ensuring consistency with the literature and focusing on the most important and frequently asked questions for families in pediatric rehabilitation practice. Questions were created through a literature review and clinical analysis and systematically divided into five categories (Appendix 1). These categories were A1 (information about CP), A2 (exercise and activity), A3 (botulinum toxin injection, serial casting, and muscle lengthening), A4 (gait and balance), and A5 (hand functions). These domains were selected to reflect the main areas of clinical management and rehabilitation in children with CP. Before submitting the questions to ChatGPT-4, the wording of the questions was revised to reflect how families typically express their questions during rehabilitation sessions. To ensure methodological consistency, the questions were written in English and entered in a random order.

Responses were generated using ChatGPT (OpenAI, San Francisco, CA, USA) via the ChatGPT web interface using the GPT-4o model available to free users in September 2025. In this study, a laptop computer (Microsoft Corp., Redmond, WA, USA) that was factory reset and had newly installed Windows 11 was used. All questions were entered independently, and previous chat history was deleted between questions to prevent contextual transfer effects. To assess response consistency, all questions were entered into ChatGPT-4 a second time under the same conditions, and the generated responses were re-evaluated and scored using the same evaluation criteria. The agreement between the two sets of responses was assessed using the intraclass correlation coefficient (ICC). As the responses suggested high consistency and conveyed similar core messages, the scores derived from the initial responses were used in the final analyses to reflect real-world user behavior, where individuals typically rely on a single generated response rather than repeating queries. Furthermore, no identifiable patient data or confidential clinical information was entered into the AI system.

### Assessment of quality, reliability, and readability

The researchers who compiled the questions also evaluated the responses; however, the scoring process was performed independently, and the evaluators were blinded to each other’s ratings. Face-to-face interviews were conducted only to resolve minor discrepancies and determine a consensus score for the final dataset; this is a commonly used approach in studies involving subjective rating scales. Readability was calculated by one researcher (RZ) using the Flesch Reading Ease formula. Questions and ChatGPT-4 responses are presented in Appendix 1. The following evaluation scales were used for scoring and analysis: the Global Quality Scale (GQS), the modified DISCERN (mDISCERN) Scale, and the Flesch Reading Ease (FRE). The characteristics of the assessment tools used are presented in Table 1.

**Table 1:** Quality, reliability, and readability measurements of ChatGPT-4 responses

Evaluation system/description/score	
**Global Quality Scale (GQS)**	
Low quality, poor flow, missing most essential information; unhelpful for patients	1
Generally poor quality; some information is present but key points are missing; limited usefulness	2
Moderate quality; some information is adequate, some is inadequate; partially useful	3
Good quality and flow; most essential information is present; helpful for patients	4
Excellent flow and quality; quite useful and comprehensive	5
**mDISCERN**	***no* = *0/yes* = *1***
1. Are the objectives clear and have they been achieved?	
2. Is the information based on reliable sources?	
3. Is the information presented balanced and unbiased?	
4. Are additional sources of information provided for the patient?	
5. Do the responses address areas of uncertainty?	
	***Total score*:**
**Flesch Reading Ease (estimated reading)**	
91–100 (Fifth grade): Very easy	
81–90 (Sixth grade): Easy	
71–80 (Seventh grade): Somewhat easy	
61–70 (Eighth-Ninth grade): Standard	
51–60 (Tenth-Twelfth grade): Somewhat difficult	
31–50 (College): Difficult	
0–30 (University graduate): Very difficult	

*mDISCERN* modified DISCERN

Interpretation of the Flesch Reading Ease Score based on Flesch readability classification [15]

The information *quality* of ChatGPT-4 responses was assessed using a 5-point GQS rating. On this scale, scores of 1 or 2 indicate low quality, 3 medium quality, and 4 or 5 high quality [13].

The *reliability* of the information provided in the responses was assessed with a modified version of DISCERN. mDISCERN is a well-established measure for assessing the reliability of health-related content. Recently, it has also been used to evaluate the reliability of AI-generated content. On the five-question scale, “yes” was scored as 1 point, and “no” as 0. Thus, the maximum score is five, and higher scores indicate greater reliability of responses. Scores were categorized as follows: 0–1 indicated low reliability, 2–3 moderate reliability, and 4–5 high reliability [14].

*Readability* was examined by calculating the FRE. The FRE score assesses text readability based on sentence length and syllable count. Scores range from 0 to 100, with higher values indicating easier readability and lower values reflecting more complex text structures. Scores of 90–100 indicate very easy, 80–89 easy, 70–79 somewhat easy, 60–69 standard, 50–59 somewhat difficult, 30–49 difficult, and 0–29 very difficult [15, 16].

### Statistical analysis

Statistical analyses were performed using the Statistical Package for the Social Sciences (SPSS), version 26.0 (IBM, Armonk, NY, USA). The normality of data distribution was assessed using both visual inspection methods (histogram plots) and statistical tests (Kolmogorov–Smirnov and Shapiro–Wilk). Continuous variables were summarized as mean (standard deviation) or median (interquartile range), depending on the distributional characteristics of the data. Variables with normal distribution were analyzed using one-way ANOVA, while variables that did not meet the normality assumption were analyzed using the Kruskal–Wallis test. A *p*-value of < 0.05 was considered statistically significant. Interrater reliability was examined by computing the ICC using a two-way random-effects model that reflects absolute agreement between evaluators. ICC was calculated for each section and for the total scores of the evaluation, as well as to assess the consistency of responses between the two time points. According to established guidelines, ICC values below 0.20 were regarded as indicating poor agreement, those between 0.21 and 0.40 as fair, 0.41 to 0.60 as moderate, 0.61 to 0.80 as good, and values exceeding 0.80 as reflecting excellent agreement.

## Result

**Fig. 1:**
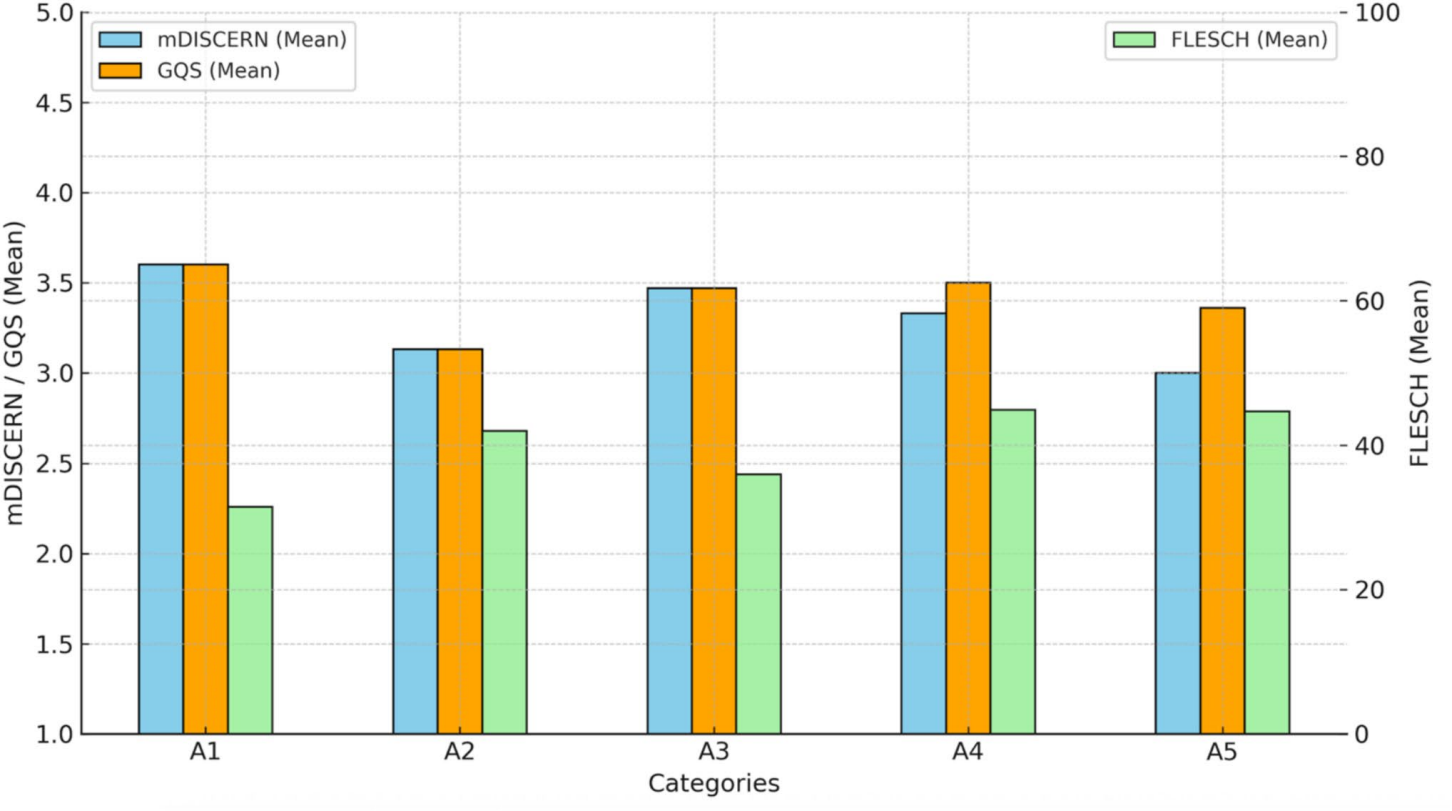
Bar chart showing reliability, quality, and readability by categories. *mDISCERN* modified DISCERN, *GQS* Global Quality Scale, *FLESCH* Flesch-Reading Ease Score

A total of 56 questions, categorized according to the relevance of the question (A1–A5), were answered using ChatGPT-4. Table 2 and Fig. 1 show the reliability (scale, 1–5), GQS (scale, 1–5), and readability (scale, 0–100) scores of the raters.

**Table 2:** Reliability, quality, and readability scores

Scores	A1 (*n* = 10)	A2 (*n* = 8)	A3 (*n* = 15)	A4 (*n* = 12)	A5 (*n* = 11)	Total score (*n* = 56)	*p value*
mDISCERN median (IQR)	4 (3–4)	3 (2.5–4)	4 (3–4)	3.5 (3–4)	3 (3–3)	3 (3–4)	0.362^a^
GQS median (IQR)	3.5 (3–4)	3 (3–3.5)	3 (3–4)	3 (3–4)	3 (3–4)	3 (3–4)	0.803^a^
FRE mean ± SD	31.47 ± 12.92	41.99 ± 10.57	35.95 ± 14.01	44.84 ± 17.39	44.68 ± 16.34	39.63 ± 2.01	0.151^b^

*mDISCERN* modified DISCERN, *GQS* Global Quality Scale, *FRE* Flesch-Reading Ease, *A *area, *A1* information about cerebral palsy, *A2* exercise and physical activity, *A3* botulinum toxin/serial casting/muscle lengthening, *A4* gait and balance, *A5* hand function, *SD* standard deviation, *IQR* interquartile range

^a^Kruskal–Wallis test

^b^One-way ANOVA

While minor differences in word choice and sentence structure were observed between the two sets of responses, the core medical content remained consistent. ICC analysis showed excellent agreement for mDISCERN and GQS scores and good agreement for FRE scores between the two sessions (ICC = 0.821 for mDISCERN (95% CI 0.696–0.895), 0.839 for GQS (95% CI 0.725–0.905), and 0.633 for FRE (95% CI 0.380–0.784)). Therefore, to maintain methodological consistency, analyses were performed using responses from the first session.

The median (IQR) values of the reliability scores assessed with the mDISCERN Scale range from 3 (3–3) (A5) to 4 (3–4) (A1). In terms of quality (GQS), the median (IQR) values range from 3 (3–3.5) (A2) to 3.5 (3–4) (A1), showing a relatively homogeneous distribution across all categories. Readability was assessed with FRE, and the mean scores ranged from 31.47 ± 12.92 to 44.84 ± 17.39. No statistically significant differences were observed between the categories. The FRE scores indicate that the answers given are difficult to read (Table 2 and Fig. 1).

The total ICC values for the mDISCERN Scale and GQS were 0.851 (95% CI 0.746–0.913) and 0.824 (95% CI 0.700–0.897), respectively (Table 3), indicating excellent agreement between the raters. The ICC values for the mDISCERN Scale ranged from 0.742 (A5; 95% CI 0.042–0.931), indicating good agreement, to 0.932 (A3; 95% CI 0.797–0.977), indicating excellent agreement. For the GQS, ICC values ranged from 0.533 (A2; 95% CI − 1.331–0.907), reflecting moderate agreement, to 0.893 (A1; 95% CI 0.569–0.973), reflecting excellent agreement.

**Table 3:** Interrater reliability analysis

		Rater 1	Rater 2	ICC (95% CI lower–upper)
A1 (*n* = 10)	mDISCERN Scale	3.50	3.60	0.899 (0.593–0.975)
	Global Quality Scale	3.60	4.00	0.893 (0.569–0.973)
A2 (*n* = 8)	mDISCERN Scale	3.13	3.38	0.750 (− 0.249–0.950)
	Global Quality Scale	3.25	3.50	0.533 (− 1.331–0.907)
A3 (*n* = 15)	mDISCERN Scale	3.33	3.73	0.932 (0.797–0.977)
	Global Quality Scale	3.47	3.47	0.851 (0.558–0.950)
A4 (*n* = 12)	mDISCERN Scale	3.17	3.25	0.785 (0.255–0.938)
	Global Quality Scale	3.25	3.50	0.860 (0.512–0.960)
A5 (*n* = 11)	mDISCERN Scale	2.91	3.27	0.742 (0.042–0.931)
	Global Quality Scale	3.27	3.36	0.724 (− 0.025–0.926)
Total score	mDISCERN Scale	3.21	3.46	0.851 (0.746–0.913)
	Global Quality Scale	3.37	3.55	0.824 (0.700–0.897)

*ICC* intraclass correlation coefficient, *CI* confidence interval, *A* area, *A1* information about cerebral palsy, *A2* exercise and physical activity, *A3* botulinum toxin/serial casting/muscle lengthening, *A4* gait and balance, *A5* hand function

*mDISCERN*, modified DISCERN

## Discussion

This study aimed to evaluate the reliability, quality, and readability of ChatGPT-4 responses to 56 frequently asked questions by families about disorders, rehabilitation strategies, and treatments seen in children with CP. The questions were divided into five categories: information about CP (A1); exercise and activity (A2); botulinum toxin injection, serial casting, and muscle lengthening (A3); gait and balance (A4); and hand functions (A5). According to the responses generated by ChatGPT-4, the highest scores were observed in the A1 category for reliability, the A1 category for quality, and the A4 category for readability; however, these differences were not statistically significant.

CP is a lifelong condition requiring continuous rehabilitation and multidisciplinary management to address motor impairments and prevent secondary complications [17]. Access to reliable treatment information and rehabilitation strategies is crucial for families and caregivers. However, regular access to a healthcare professional may not always be possible for every individual and family. Therefore, AI-powered tools like ChatGPT-4 are becoming increasingly important, especially for those with limited time, transportation, or financial resources. Patients and their families frequently use ChatGPT-4 to explain received reports and prescriptions, as well as to search for alternative treatment options, investigate risks, and get answers to questions [9]. While online information can be an easy way to find information about CP, variations in the quality and understandability of the information available can cause further confusion and problems for parents.

Studies evaluating the quality and reliability of information produced by AI-based systems for various disease groups yield inconsistent findings. ChatGPT reportedly has promising applications when used under medical supervision in pediatric healthcare systems and can produce accurate patient education materials [18], but responses can be complex or occasionally inaccurate [19]. ChatGPT-4 suggested high accuracy in diagnostic reasoning and triage decisions for critical pediatric illnesses; however, treatment plans were found to be lacking and included risky recommendations [20]. The study evaluating ChatGPT-4 responses regarding physical activity in children with cystic fibrosis found that the responses provided moderate to high levels of reliability and quality [21]. Uzun Bektaş et al. showed that ChatGPT-4 and LLaMA 3.1 provided moderate reliability and high-quality answers to clinician-generated questions on familial Mediterranean fever [22]. In our study, the reliability and quality of ChatGPT-4 responses were found to be moderate to good. The reliability scores had a homogeneous distribution with median values generally between 3 and 4. A study by Temel et al. reported that only 29.6% of human-developed online educational materials related to cerebral palsy were of high quality, with the majority classified as low quality [23]. In a study evaluating ChatGPT-4 responses for CP, ten disease-related questions were asked to ChatGPT-4. The average reliability and usefulness of ChatGPT responses to the ten questions indicated that it was generally a reliable and partially useful source of information for most of the questions. However, it also noted the limitations of the information presented, suggesting caution in such areas [24]. In contrast, our study included 56 questions divided into five categories specific to treatment and rehabilitation strategies, and reliability was assessed using the mDISCERN Scale. ChatGPT-4 responses in our study were of moderate reliability and quality, consistent with previous studies. AI programs typically do not provide source citations in their answers. Two questions from the mDISCERN Scale, which require source citation, suggested this deficiency in the responses generated by ChatGPT-4. This shows that the content mostly conveys basic medical information accurately, but source citation is inadequate, scientific references are missing, and impartiality/updatedness is limited.

Our study findings indicate that ChatGPT-4’s CP-related responses are of moderate-to-good reliability and quality. However, the model’s responses varied in terms of reliability and quality scores depending on the question type (no statistically significant difference). Questions containing general information (A1) were rated as more accurate, informative, and of higher quality, and tended to receive higher scores. For example, answers to questions about general knowledge of the disease, such as “What is cerebral palsy?” in category A1, received more reliable and higher quality scores. However, some responses may vary depending on evolving clinical knowledge and context. For instance, while ChatGPT-4 indicated that genetic testing is rarely performed in the diagnosis of cerebral palsy, genetic factors are increasingly recognized, and genetic testing is now more frequently used in many clinical settings. Similarly, some factors mentioned as causes of cerebral palsy (e.g., low birth weight) may be more accurately considered risk factors rather than definitive causes. Patient-specific activity questions received lower scores (A2, A5). These included questions asking about personalized exercise programs and activity examples, such as “My child with cerebral palsy can stand. What types of exercises are appropriate for them?” and “How can my child with cerebral palsy use their hands more effectively during daily activities?”. These findings suggest that ChatGPT-4 may provide general information about cerebral palsy; however, its responses may be limited, particularly when addressing highly individualized or patient-specific issues. General and/or passive motor interventions recommended by ChatGPT-4 are less effective in improving function and movement in children with CP [25]. Therefore, experts generally recommend practicing real-life tasks and activities using spontaneous, active movements [25]. In addition, in several responses, ChatGPT-4 appropriately recommended consulting healthcare professionals for further evaluation and guidance. Developing LLMs with input and clinical experience from expert researchers in the field may help improve the content quality of artificial intelligence tools. In this context, this study contributes to the growing body of research evaluating health information generated by AI. The use of commonly employed assessment tools to examine the quality, reliability, and readability of ChatGPT-4 responses may also guide future studies investigating the effectiveness of AI-based models in rehabilitation and healthcare.

Readability studies have reported that for patients to be able to easily read and understand information, their reading level should be at or below a 6th-grade level (FRE score 80–90) [26, 27]. In a study, the readability levels of CP-related online materials were reported to exceed recommended thresholds, indicating that much of the content may not be easily understandable for patients and their families [23]. A study evaluating ChatGPT-4’s responses on Scoliosis showed that readability levels were predominantly university-level [28]. Similarly, a study investigating the readability of ChatGPT-4 responses regarding physical activity in children with cystic fibrosis reported a mean FRE score of 38.07. This suggests that despite high readability, the responses were not consistent with age and reading level [21]. Readability assessments of our study indicated that the model required university-level understanding to interpret its responses. The mean FRE scores ranged from 31.47 ± 12.92 to 44.84 ± 17.39, indicating that the texts were difficult to read and understand. This readability level suggests that the information provided may not be easily understandable for parents of children with CP, who represent a heterogeneous group in terms of education and health literacy. The use of complex language and medical terminology may limit the accessibility of online materials, potentially hindering parents’ ability to comprehend and apply the information effectively in home-based care and rehabilitation. Therefore, simplifying the language and improving readability may help increase the accessibility of such resources for families.

While variability in LLMs’ outcomes is a known issue [28], our findings revealed a high degree of agreement among repeated responses, as suggested by the ICC. This shows that responses generated under the same conditions can consistently reveal the basic information. From a clinical perspective, this consistency is important because inconsistent outcomes can potentially impact the reliability of the information accessed by users. The agreement observed in this study supports the reliability of responses assessed in terms of overall quality and content consistency. However, it must be acknowledged that some variability may still exist, particularly in more complex or individualized questions. In addition, as LLMs are continuously updated, responses may vary across different model versions over time, which may limit reproducibility. Therefore, while the findings suggest that single-response outcomes can reasonably reflect the information users encounter in real-world user behavior, future studies could examine response variability in more detail across different prompts, contexts, and repeated interactions to better understand their impact on clinical decision-making and patient education.

## Strengths and limitations

To our knowledge, this is the first study to evaluate the reliability, quality, and readability of information provided by AI-powered language models such as ChatGPT-4 regarding rehabilitation strategies in children with CP. The questions were intended to represent commonly discussed domains in CP rehabilitation rather than country-specific information needs. Another strength of the study is that the questions were pre-categorized by the researchers before being submitted to ChatGPT-4, which enabled a more organized assessment and facilitated a structured analysis of the responses. However, there are several limitations to our study. First, ChatGPT-4 may respond differently depending on model updates or version changes, as large language models are continuously evolving. In this study, responses were generated using the free web-based version of ChatGPT-4 available at the time of data collection. Therefore, the findings may vary with future model updates or different platform versions, which should be considered when interpreting the results. Second, all questions on ChatGPT-4 are written in English. Questions entered in a different language may also receive different answers. In future studies, the reliability of the answers given to questions asked in different languages can be examined. Third, the evaluation of the responses relied on the clinical judgement of the two evaluators, which may represent a potential limitation. In addition, some clinical topics may be interpreted differently depending on evolving scientific evidence and local clinical practices, which may influence how the accuracy of certain responses is perceived. Finally, the question categories are not structured according to established frameworks such as the International Classification of Functioning, Disability and Health (ICF) or the F-words framework. The questions were instead grouped based on common topics encountered in clinical treatment and rehabilitation practice. Future studies may consider using these frameworks to structure question domains more systematically.

## Conclusion

The present study suggests that ChatGPT-4 responses related to treatment and rehabilitation strategies for children with cerebral palsy showed moderate levels of reliability and generally acceptable quality. In particular, responses to questions addressing theoretical aspects of the condition, such as its etiology and progression, tended to show relatively higher reliability and quality. Individualized exercise programs and activity recommendations can be more diverse and reliable under the supervision of experts. Furthermore, the responses provided by ChatGPT-4 were difficult to read and required a university-level education to understand. Because families of children with CP may have varying educational backgrounds, some may find the answers difficult to understand. Therefore, further improvements in language simplicity and clarity may help enhance the understandability of the model’s responses. In disorders that impact multiple developmental domains, such as CP, ChatGPT-4’s informative responses may be more useful when used under the supervision of experts and clinicians.

## Supplementary Information

Below is the link to the electronic supplementary material.

ESM 1 (PDF 559 KB)

## Data Availability

Data supporting the findings of this study are available from the corresponding author upon reasonable request.

## Abbreviations

AI: Artificial intelligence
CP: Cerebral palsy
LLM: Large language model
GQS: Global Quality Scale
FRE: Flesch Reading Ease Scale

## References

[1] Dlamini MD, Chang YJ, Nguyen TTB (2023) Caregivers’ experiences of having a child with cerebral palsy: a meta-synthesis. J Pediatr Nurs 73:157–168. 10.1016/j.pedn.2023.08.026 37690430 10.1016/j.pedn.2023.08.026

[2] Galea C et al (2019) Cerebral palsy trends in Australia (1995–2009): a population-based observational study. Dev Med Child Neurol 61(2):186–193. 10.1111/dmcn.14011 30187914 10.1111/dmcn.14011

[3] Khandaker G et al (2019) Epidemiology of cerebral palsy in Bangladesh: a population-based surveillance study. Dev Med Child Neurol 61(5):601–609. 10.1111/dmcn.14013 30394528 10.1111/dmcn.14013

[4] Patel DR et al (2020) Cerebral palsy in children: a clinical overview. Transl Pediatr 9(Suppl 1):S125. 10.21037/tp.2020.01.01 10.21037/tp.2020.01.01 PMC7082248 32206590

[5] Jones RA et al (2011) Promoting fundamental movement skill development and physical activity in early childhood settings: a cluster randomized controlled trial. Pediatr Exerc Sci 23(4):600–615. 10.1123/pes.23.4.600 22109783 10.1123/pes.23.4.600

[6] Wati RS, Purwati NH, Permatasari TAE (2020) Caring for cerebral palsy children: Indonesian parents’ experience. Int J Soc Sci World 2(2):117–121

[7] Gimigliano F, Negrini S (2017) The World Health Organization “Rehabilitation 2030: a call for action”. Eur J Phys Rehabil Med 53(2):155–168. 10.23736/s1973-9087.17.04746-3 10.23736/S1973-9087.17.04746-3 28382807

[8] Blackman JA, Conaway MR (2014) Adolescents with cerebral palsy: transitioning to adult health care services. Clin Pediatr 53(4):356–363. 10.1177/0009922813510203 10.1177/0009922813510203 24275216

[9] Giorgino R et al (2023) ChatGPT in orthopedics: a narrative review exploring the potential of artificial intelligence in orthopedic practice. Front Surg 10:1284015. 10.3389/fsurg.2023.1284015 38026475 10.3389/fsurg.2023.1284015 PMC10654618

[10] Das KP, Chandra J (2023) A survey on artificial intelligence for reducing the climate footprint in healthcare. Energy Nexus 9:100167. 10.1016/j.nexus.2022.100167

[11] Haug CJ, Drazen JM (2023) Artificial intelligence and machine learning in clinical medicine. N Engl J Med 388(13):1201–1208. 10.1056/NEJMra2302038 36988595 10.1056/NEJMra2302038

[12] Parviainen J, Rantala J (2022) Chatbot breakthrough in the 2020s? An ethical reflection on the trend of automated consultations in health care. Med Health Care Philos 25(1):61–71. 10.1007/s11019-021-10049-w 34480711 10.1007/s11019-021-10049-w PMC8416570

[13] Bernard A et al (2007) A systematic review of patient inflammatory bowel disease information resources on the World Wide Web. Am J Gastroenterol 102(9):2070–2077. 10.1111/j.1572-0241.2007.01325.x 17511753 10.1111/j.1572-0241.2007.01325.x

[14] Charnock D et al (1999) DISCERN: an instrument for judging the quality of written consumer health information on treatment choices. J Epidemiol Community Health 53(2):105–111. 10.1136/jech.53.2.105 10396471 10.1136/jech.53.2.105 PMC1756830

[15] Flesch R (1948) A new readability yardstick. J Appl Psychol 32(3):221–233. 10.1037/h0057532 18867058 10.1037/h0057532

[16] Jindal P, MacDermid JC (2017) Assessing reading levels of health information: uses and limitations of Flesch formula. Educ Health 30(1):84–88. 10.4103/1357-6283.210517 10.4103/1357-6283.210517 28707643

[17] Rosenbaum P, Paneth N, Leviton A, Goldstein M, Bax M, Damiano D, Jacobsson B (2007) A report: the definition and classification of cerebral palsy April 2006. Dev Med Child Neurol Suppl 109(suppl 109):8–14 17370477

[18] Zhu S, Xie Y, Tang Y, Yu Z, Zhao R, Dong X (2025) New chapter in pediatric medicine: technological evolution, application, and evaluation system of large language models. Eur J Pediatr 184(12):809. 10.1007/s00431-025-06602-x 41324732 10.1007/s00431-025-06602-x

[19] Douma H et al (2025) Leveraging ChatGPT to strengthen pediatric healthcare systems: a systematic review. Eur J Pediatr 184(8):478. 10.1007/s00431-025-06320-4 40650728 10.1007/s00431-025-06320-4

[20] Tausky O et al (2025) Large language model as a clinical decision support tool in the initial management of critically ill children: a pilot evaluation. Eur J Pediatr 184(12):757. 10.1007/s00431-025-06630-7 41238850 10.1007/s00431-025-06630-7

[21] Çelik Z, Sari F (2025) Evaluation of ChatGPT-4 responses on physical activity guidance in children with cystic fibrosis: reliability, quality, and readability. Eur J Pediatr 184(11):676. 10.1007/s00431-025-06488-9 41076450 10.1007/s00431-025-06488-9

[22] Uzun Bektaş A, Bora B, Ünsal E (2025) Comparative evaluation of ChatGPT and LLaMA for reliability, quality, and accuracy in familial Mediterranean fever. Eur J Pediatr 184(8):491. 10.1007/s00431-025-06318-y 40679644 10.1007/s00431-025-06318-y

[23] Temel MH, Batıbay S, Bağcıer F (2023) Quality and readability of online information on cerebral palsy. J Consum Health Internet 27(3):266–281. 10.1080/15398285.2023.2235531

[24] Ata AM et al (2024) Evaluation of informative content on cerebral palsy in the era of artificial intelligence: the value of ChatGPT. Phys Occup Ther Pediatr 44(5):605–614. 10.1080/01942638.2024.2316178 38361368 10.1080/01942638.2024.2316178

[25] Novak I et al (2020) State of the evidence traffic lights 2019: systematic review of interventions for preventing and treating children with cerebral palsy. Curr Neurol Neurosci Rep 20(2):3. 10.1007/s11910-020-1022-z 32086598 10.1007/s11910-020-1022-z PMC7035308

[26] Cotugna N, Vickery CE, Carpenter-Haefele KM (2005) Evaluation of literacy level of patient education pages in health-related journals. J Community Health 30(3):213–219. 10.1007/s10900-004-1959-x 15847246 10.1007/s10900-004-1959-x

[27] Eltorai AE et al (2014) Readability of patient education materials on the American Association for the Surgery of Trauma website. Arch Trauma Res 3(2):e18161. 10.5812/atr.18161 25147778 10.5812/atr.18161 PMC4139691

[28] Çıracıoğlu AM, Dal Erdoğan S (2025) Evaluation of the reliability, usefulness, quality, and readability of ChatGPT’s responses on scoliosis. Eur J Orthop Surg Traumatol 35(1):123. 10.1007/s00590-025-04198-4 40100428 10.1007/s00590-025-04198-4

