# Supplementary material for: Evaluation of the quality, reliability, and readability of ChatGPT-4 responses related to the treatment and rehabilitation of children with cerebral palsy
Source: Eur J Pediatr. 2026 Apr 24;185(5):297. doi: 10.1007/s00431-026-06979-3 (PMC13109198; doi:10.1007/s00431-026-06979-3)
Supplement: Supplementary file 1 — (PDF 559 KB) [file 431_2026_6979_MOESM1_ESM.pdf]

This appendix material presents the full set of questions (prompts) used in the study. All questions (prompts) were entered as plain text without any modifications, additional system-level instructions, or prompt techniques. Each question (prompt) was submitted independently in a separate session, and chat history was cleared between sessions to prevent contextual carryover effects. All interactions were conducted using the default settings of the platform available at the time of data collection. These procedures were applied to ensure consistency and independence of responses across all queries

A1: Information about the CP

1. What is cerebral palsy?
2. What causes cerebral palsy?
3. How is cerebral palsy diagnosed?
4. Does cerebral palsy worsen over time?
5. Can cerebral palsy be completely cured with treatment?
6. What are the different types of cerebral palsy?
7. What does spasticity mean?
8. What can be done to reduce spasticity?
9. How does cerebral palsy affect bones and joints?
10. Does cerebral palsy affect my child's vision and hearing?

A2: Exercise and Physical Activity

1. My child with cerebral palsy can stand. What types of exercises are appropriate for them?
2. What exercises can my child with cerebral palsy perform while using a wheelchair?
3. Does exercise reduce spasticity in children with cerebral palsy?
4. How should I adjust exercise intensity for my child with cerebral palsy, and how many days per week should they exercise?
5. Are there home-based activities that my child with cerebral palsy can safely perform?
6. What types of equipment should I use when exercising with my child who has cerebral palsy?
7. Can my child with cerebral palsy participate in physical education classes at school?
8. How does exercise affect the mental health of my child with cerebral palsy?

A3: Botulinum Toxin / Serial Casting / Muscle Lengthening

1. I have a child with cerebral palsy. What is botulinum toxin (Botox), and does it reduce spasticity?
2. Does Botox cause any harm to the muscles in children with cerebral palsy?
3. What is the maximum number of times Botox can be injected into the same muscle in a child with cerebral palsy?
4. Is it safe for my child with cerebral palsy to perform exercises after receiving botulinum toxin injections?
5. What activities can enhance the therapeutic effects of botulinum toxin in my child with cerebral palsy?
6. When should I begin stretching exercises after my child with cerebral palsy receives botulinum toxin?
7. What is the duration of botulinum toxin’s effect in children with cerebral palsy?
8. Under what circumstances is serial casting used for children with cerebral palsy?
9. How is serial casting performed in children with cerebral palsy?
10. Can botulinum toxin injections and serial casting be used together in my child with cerebral palsy?
11. Which exercises should be performed after serial casting is completed in my child with cerebral palsy?
12. At what age can muscle lengthening surgery be performed for calf muscles in children with cerebral palsy?
13. What should I pay attention to after muscle lengthening surgery in my child with cerebral palsy?
14. What exercises should be done after calf muscle lengthening surgery for my child with cerebral palsy?
15. What is Selective Percutaneous Myofascial Lengthening (SPML)?

A4: Gait and Balance

1. How does cerebral palsy affect my child’s walking ability?
2. Are orthotic devices necessary to support walking in my child with cerebral palsy?
3. How does my child’s motor function level influence their gait pattern?
4. What can be done for my child with cerebral palsy who walks on their toes?
5. Why does my child with cerebral palsy experience knee hyperextension while walking?
6. Can I help improve my child’s walking speed?
7. My child with cerebral palsy is able to walk. What type of footwear should I choose?
8. What exercises can I do to improve my child’s balance?
9. What activities are appropriate for children with cerebral palsy who are unable to walk?
10. What technological or game-based equipment can help improve walking and balance in my child with cerebral palsy?

11. Does hippotherapy improve gait and balance in children with cerebral palsy?

12. What innovative approaches can help enhance gait and balance in my child with cerebral palsy?

A5: Hand Function

1. How are hand skills affected in my child with cerebral palsy?
2. Is it possible to improve my child's gross and fine motor skills if they have cerebral palsy?
3. How can my child with cerebral palsy use their hands more effectively during daily activities?
4. How can reduced grip strength be improved in my child with cerebral palsy?
5. What activity suggestions are appropriate for tasks performed with one hand in children with cerebral palsy?
6. How can bimanual (two-handed) activities be improved in my child with cerebral palsy?
7. Are there home-based hand exercises I can do with my child who has cerebral palsy?
8. Is restricting the more frequently used hand beneficial for encouraging the use of the less-used hand in my child with cerebral palsy?
9. What strategies can help teach a new hand skill to my child with cerebral palsy?
10. Is it appropriate to use devices that help keep the hand open in children with cerebral palsy?
11. How can technology be used to improve hand and arm function in my child with cerebral palsy?

**Questions-Categories**	**The list of questions**	**ChatGPT-4's answers**
**1-A4**	**What activities are appropriate for children with cerebral palsy who are unable to walk?**	Children with cerebral palsy who are unable to walk can still participate in a wide range of activities that promote physical, cognitive, and social development. The selection of activities should be individualized based on the child's motor abilities, interests, and recommendations from healthcare professionals.<br>**1. Sensory and Table-Top Activities**<br>Activities such as drawing, painting, playing with modeling clay, building blocks, and engaging with sensory bins (e.g., rice, sand, or water-based materials) support fine motor skills, sensory processing, and cognitive engagement.<br>**2. Music-Based Interventions**<br>Music activities, including the use of simple percussion instruments, singing, or rhythmic exercises performed in a seated position, can enhance motor coordination, attention, and emotional expression.<br>**3. Seated Physical Exercises**<br>Seated stretching routines, ball-handling tasks (e.g., passing, rolling, or catching a ball), and core-stability exercises can improve strength, balance, and postural control. These activities should be conducted under the guidance of a physical therapist.<br>**4. Adaptive or Supported Sports**<br>Children may participate in adapted sports such as seated basketball, adaptive bowling, or swimming with appropriate supervision and specialized equipment. Aquatic therapy is often particularly beneficial due to reduced joint stress and increased freedom of movement in water.<br>**5. Nature-Based and Low-Intensity Outdoor Activities**<br>Outdoor experiences, including wheelchair-accessible nature walks, bird watching, or simple play activities such as blowing bubbles, provide sensory stimulation and opportunities for social interaction.<br>**6. Social and Educational Games**<br>Reading activities, educational board games, and imaginative play scenarios (e.g., role-playing) promote language development, cognitive skills, and social engagement.
**2-A2**	**My child with cerebral palsy can stand. What types of exercises are appropriate for them?**	**1. Standing and Balance Activities**<br>Supported standing at a table or couch<br>Gentle weight shifting while holding support<br>Reaching for toys in standing to improve trunk control<br>**2. Early Strengthening Activities**<br>Assisted sit-to-stand from a caregiver's lap or low seat<br>Supported mini-squats while holding onto stable furniture<br>Pull-to-stand practice using a sturdy surface<br>**3. Mobility and Pre-Walking Activities**<br>Cruising along furniture<br>Supported stepping with hand-hold assistance<br>Short bouts of walking using a pediatric walker (if recommended)<br>**4. Coordination and Play-Based Activities**<br>Rolling or passing a soft ball while standing with support<br>Placing toys slightly out of reach to encourage controlled movement<br>Simple push toys or walkers under supervision

**3-A4**	**How does cerebral palsy affect my child's walking ability?**	Cerebral palsy (CP) can affect a child's walking ability in several ways, depending on which areas of the brain are involved and the severity of motor impairments.<br>**1. Muscle Tone Differences**<br>**High muscle tone (spasticity)** can make the legs stiff and limit smooth movement.<br>**Low muscle tone** can make it harder to maintain posture and balance.<br>**2. Muscle Weakness**<br>Weakness in the hips, legs, or trunk can affect stability and make standing and stepping more difficult.<br>**3. Coordination and Motor Control**<br>Children may have difficulty coordinating movements, leading to slower, less fluid walking patterns.<br>**4. Balance Challenges**<br>CP can impact balance systems, making it harder to stand, shift weight, or walk without support.<br>**5. Joint and Bone Changes**<br>Over time, muscle tightness may contribute to joint limitations or altered walking patterns.<br>If you'd like, I can also provide a **brief list of early signs** or **ways to support walking development** for your child's age.
**4-A5**	**How can technology be used to improve hand and arm function in my child with cerebral palsy?**	Technology can play a powerful role in improving hand and arm function in children with cerebral palsy by enhancing motivation, supporting motor learning, and providing targeted, repetitive practice. Several types of technological tools are commonly used in pediatric rehabilitation:<br>**1. Functional electrical stimulation (FES):**<br>FES delivers small electrical impulses to specific muscles in the hand or arm to help the child practice opening the hand, extending the wrist, or improving reach-and-grasp. It can strengthen weak muscles, reduce the effects of spasticity, and reinforce more coordinated movement patterns.<br>**2. Robotic and electromechanical devices:**<br>Robotic arm trainers or assistive exoskeletons provide guided, repetitive movement practice that helps children learn smoother reaching, lifting, and grasping motions. They can adjust the level of support based on the child's ability, making therapy more individualized and intensive.<br>**3. Virtual reality (VR) and interactive gaming systems:**<br>VR environments and motion-controlled games encourage purposeful upper-limb movement in a fun, engaging way. These systems help improve range of motion, reaction time, bilateral arm use, and hand–eye coordination while motivating the child to practice repeatedly.<br>**4. Computer-based training and tablet apps:**<br>Apps that respond to touch, swipe, or fine-motor gestures are used to work on finger isolation, grasp–release skills, and precision. Many programs allow therapists to tailor activities to target specific movement goals.<br>**5. Adaptive equipment and smart orthoses:**<br>Dynamic hand splints, powered grasp-assist gloves, and other wearable devices can help maintain an open hand position or support functional grasp during daily activities. Some newer devices incorporate sensors that provide feedback about movement quality.<br>**6. Motion-tracking and biofeedback systems:**<br>These tools use sensors to show the child how their muscles are working in real time, helping them learn to reduce unwanted movements, activate specific muscles, and refine motor control.<br>Because each child's motor abilities and goals are unique, the most effective technologies are chosen and monitored by a pediatric occupational therapist or rehabilitation specialist. When integrated into a comprehensive therapy program, these tools can significantly enhance hand and arm function, independence, and confidence.

**5-A3**	**What is Selective Percutaneous Myofascial Lengthening (SPML)?**	**Selective Percutaneous Myofascial Lengthening (SPML)** is a minimally invasive surgical technique used primarily to treat **muscle contractures** and **spasticity**, most commonly in children with **cerebral palsy**, but also in adults with similar movement-limiting conditions.<br><br>**What SPML Involves**<br><br>• The surgeon makes **very small skin incisions** (often just a few millimeters).<br>• Using specialized instruments, they **release tight fascia** (the connective tissue around muscles) at targeted points.<br>• The muscle itself is usually **not fully cut**, unlike in traditional muscle-lengthening surgery.<br>• The goal is to allow **greater range of motion** while minimizing trauma.<br><br>**Why It's Done**<br><br>• Toe walking<br>• Hamstring tightness<br>• Hip adductor tightness<br>• Contractures that limit mobility or hamper function<br><br>**Benefits Often Reported**<br><br>• Smaller incisions → **less scarring**<br>• **Shorter recovery** time compared to open surgery<br>• Performed as **outpatient surgery** in many cases<br>• Can improve gait and comfort when combined with therapy<br><br>**Risks or Considerations**<br><br>• Results vary depending on the patient's underlying condition.<br>• Requires **intensive postoperative therapy** (often serial casting, stretching, gait training).<br>• May need to be repeated if tightness recurs.<br>• Long-term outcomes continue to be evaluated; high-quality comparative studies are limited.
**6-A5**	**Is it appropriate to use devices that help keep the hand open in children with cerebral palsy?**	Using devices designed to help keep the hand open **can be appropriate** for children with cerebral palsy, but their use depends on the child's specific motor profile, goals, and tolerance.<br><br>In clinical practice, hand-opening devices (such as soft splints, functional hand orthoses, or dynamic extension aids) are considered when a child has **increased flexor tone**, difficulty extending the fingers, or a tendency to keep the hand fisted, which may limit grasp–release function, hygiene, or comfort. These devices can help maintain a more functional hand position, support joint alignment, and reduce the risk of secondary contractures. They are sometimes used during functional tasks to promote improved hand opening or as part of a therapy plan that includes stretching, sensory input, and task-specific practice.<br><br>However, the appropriateness of these devices is **highly individualized**. Overly rigid or prolonged positioning can interfere with active motor learning or exacerbate abnormal movement patterns. Dynamic or adjustable designs are often preferred because they allow the child to actively attempt extension rather than being fully immobilized. It is also essential to consider skin integrity, the child's sensory tolerance, and whether the device supports—not restricts—functional engagement.<br><br>Overall, these devices can be beneficial when used with clear goals and under the guidance of an occupational therapist or pediatric rehabilitation specialist who can ensure proper fit, safe use, and integration into a broader therapeutic program.
**7-A4**	**What innovative approaches**	Innovative approaches to improving gait and balance in children with cerebral palsy increasingly combine traditional physiotherapeutic methods with technology-enhanced, task-oriented interventions. Intensive, motor-learning–based gait training helps strengthen

	**can help enhance gait and balance in my child with cerebral palsy?**	neuromuscular pathways by repeatedly practicing functional movements relevant to daily activities. Virtual reality and interactive, game-based therapies provide enriched sensory environments that can enhance motivation while promoting postural control and adaptive stepping strategies. Robotic gait trainers and pediatric exoskeletons enable safe, repetitive practice of more efficient gait patterns, while functional electrical stimulation (FES) supports timely muscle activation, particularly in controlling the ankle and knee during ambulation. Additional modalities such as dynamic balance platforms, vestibular stimulation exercises, aquatic therapy, and hippotherapy can further improve postural stability and coordination. Because the motor profile and functional goals vary widely among children with cerebral palsy, individualized assessment and multidisciplinary planning are essential for selecting and optimizing the most appropriate intervention strategies.
**8-A3**	**Under what circumstances is serial casting used for children with cerebral palsy?**	Serial casting is typically used for children with cerebral palsy when there is a need to gradually increase passive range of motion and reduce musculoskeletal impairments caused by spasticity-related muscle shortening. It is most commonly indicated in cases of dynamic or mild-to-moderate fixed contractures, particularly in the ankle plantarflexors, hamstrings, or forearm musculature, where conservative interventions such as stretching and strengthening have not produced sufficient improvement. Serial casting is also employed after botulinum toxin injections to maximize neuromuscular lengthening during the period of reduced spasticity, and it can be part of a pre-surgical or post-surgical management plan to maintain alignment and prevent recurrence of deformity. Additionally, it may be used when improved joint alignment is needed to facilitate safer and more efficient gait patterns, enhance orthotic fit, or prevent secondary complications such as pain, skin breakdown, or progressive contracture. Careful monitoring is essential, as casting is contraindicated in the presence of significant fixed deformity, skin integrity issues, vascular compromise, or poor tolerance to immobilization.
**9-A3**	**How is serial casting performed in children with cerebral palsy?**	Serial casting for children with cerebral palsy is a therapeutic technique used to gradually improve range of motion—most often in joints affected by spasticity, such as the ankle (equinus deformity), knee, or elbow. The process is typically led by a physical therapist or rehabilitation specialist and focuses on gently stretching tight muscles over time. Before the first cast is applied, the clinician assesses the child's muscle tone, joint mobility, functional goals, and any contraindications such as skin breakdown, fractures, or vascular concerns. The limb is then positioned in a tolerable stretch, and soft padding is placed to protect bony prominences and maintain skin integrity.<br><br>Once positioned, a fiberglass or plaster cast is applied to hold the limb in the targeted stretch. The cast remains on for several days—commonly 5 to 7—allowing the muscles to adapt gradually to the new lengthened position. When the cast is removed, range of motion is reassessed, and a new cast is applied with the joint placed in a slightly greater stretch than before. This process repeats over several weeks, and it often involves three to six successive casts, depending on the severity of the contracture and the child's tolerance.<br><br>Throughout serial casting, clinicians monitor the child closely for signs of discomfort, skin irritation, swelling, or impaired circulation. Parents are educated about observing the toes or fingers for color changes, ensuring the cast remains dry, and reporting any concerns. After the casting series is completed, the improved range of motion is maintained with ongoing stretching, strengthening, physical therapy exercises, orthotic use, and functional training. This comprehensive approach helps maximize the long-term benefits of serial casting and supports improved mobility, gait, and participation in daily activities.
**10-A1**	**What is cerebral palsy?**	Cerebral palsy (CP) is a group of permanent disorders that affect movement, posture, and muscle coordination due to abnormal development of the brain or damage to the developing brain, usually before birth, during birth, or shortly after. It is non-progressive, meaning the original brain injury does not worsen over time, though the effects on the body may change as a child grows.<br>Children with cerebral palsy may experience muscle stiffness (spasticity), involuntary movements, poor balance, or difficulties with fine motor skills. The condition can also be associated with other challenges, such as speech or swallowing difficulties, vision or hearing impairments, intellectual disability, or seizures, though not all children have these additional issues. CP varies widely in severity—some children may walk independently, while others may need assistive devices or wheelchairs.<br>Overall, cerebral palsy results from disruptions to normal brain development, and early intervention with therapies such as physical therapy, occupational therapy, speech therapy, orthotics, and medical treatments can significantly improve function, independence, and quality of life.
**11-A4**	**Does hippotherapy improve gait**	Yes, research generally supports that hippotherapy—the clinical use of horseback movement by trained therapists—can improve gait, balance, postural control, and overall motor function in many children with cerebral palsy.

	**and balance in children with cerebral palsy?**	In hippotherapy, the horse's rhythmic, three-dimensional movement provides sensory and motor input similar to the human walking pattern. This challenges the child's trunk, pelvis, and postural muscles, helping to improve core stability, dynamic balance, and coordination. Because the horse provides consistent yet variable movement, children practice continual adjustments that can translate into better control when standing or walking.<br><br>Studies over the past two decades have shown that hippotherapy can lead to improvements such as increased stride length, better symmetry, improved gross motor function, and enhanced sitting or standing balance. The greatest benefits tend to occur when hippotherapy is used as part of a broader therapy plan, including physical therapy and strengthening exercises. Effects may vary depending on the child's type of cerebral palsy, level of motor impairment, frequency of sessions, and the therapist's expertise.<br><br>While hippotherapy is not a cure, it is considered a valuable adjunct to traditional rehabilitation and is often reported by families and clinicians to enhance motivation, engagement, and confidence along with physical outcomes.
**12-A1**	**How is cerebral palsy diagnosed?**	Cerebral palsy (CP) is diagnosed primarily through **clinical evaluation**, as there isn't a single test that can definitively confirm it. The process usually involves multiple steps to assess motor function, developmental history, and rule out other conditions.<br>**1. Medical History**<br>• **Pregnancy and birth history:** Premature birth, low birth weight, lack of oxygen during delivery, infections, or exposure to toxins can increase risk.<br>• **Developmental milestones:** Delays in rolling over, sitting, crawling, or walking may indicate motor problems.<br>**2. Physical and Neurological Examination**<br>• **Muscle tone and posture:** Doctors look for **spasticity (stiff muscles)**, **hypotonia (floppy muscles)**, or mixed types.<br>• **Reflexes:** Persistent infant reflexes (like the Moro reflex) beyond the typical age can be a sign.<br>• **Movement patterns:** Asymmetry in movement, involuntary movements, or poor coordination are assessed.<br>• **Motor skills:** Fine and gross motor skills are tested to check for abnormalities.<br>**3. Developmental Assessment**<br>• Standardized tools may be used to evaluate **motor, cognitive, and social development** relative to age expectations.<br>**4. Imaging Studies**<br>While imaging **cannot confirm CP**, it helps identify brain abnormalities:<br>• **MRI (Magnetic Resonance Imaging):** Detects brain malformations, white matter injury, or damage consistent with CP.<br>• **CT scans:** Less commonly used but can show structural changes.<br>• **Ultrasound:** Sometimes used for infants, especially preterm babies.<br>**5. Laboratory Tests**<br>• Rarely, blood tests or genetic testing may be done to **rule out metabolic or genetic disorders** that mimic CP.<br><br>**6. Observation Over Time**<br>• CP is often diagnosed over months or years as doctors observe **how the child grows and develops motor skills**.<br>• Early diagnosis is possible, especially in high-risk infants, sometimes as early as **12–18 months**.
**13-A1**	**What causes cerebral palsy?**	Cerebral palsy (CP) is a group of disorders that affect a person's movement, muscle tone, and posture. It is caused by **damage to the developing brain**, usually before, during, or shortly after birth. The damage disrupts the brain's ability to control movement and coordination.<br>1. **Prenatal (before birth) causes** – the most common:<br>○ **Abnormal brain development** in the womb.<br>○ **Lack of oxygen (hypoxia) or blood flow** to the baby's brain.<br>○ **Maternal infections** during pregnancy (e.g., rubella, cytomegalovirus).<br>○ **Exposure to toxins** or drugs during pregnancy.<br>○ **Genetic mutations** affecting brain development.<br>2. **Perinatal (around birth) causes**:

		○ **Birth complications** leading to oxygen deprivation, such as difficult labor or premature separation of the placenta.<br>○ **Premature birth**—the earlier a baby is born, the higher the risk.<br>○ **Low birth weight**.<br>3. **Postnatal (after birth, up to ~2 years)**:<br>○ **Severe infections** like meningitis or encephalitis.<br>○ **Head injuries** or trauma.<br>○ **Stroke** in infancy.<br>○ **Severe jaundice** (kernicterus) if untreated.<br>It's important to note that in many cases, the exact cause of cerebral palsy is **unknown**, and it may result from a combination of factors.
**14-A5**	**How are hand skills affected in my child with cerebral palsy?**	In children with **cerebral palsy (CP)**, hand skills are often affected due to the underlying motor impairments associated with the condition. The effects vary depending on the type and severity of CP, but generally involve challenges with **muscle tone, coordination, and control**.<br>**1. Types of Hand Skill Difficulties**<br>Hand skills can be affected in several ways:<br>**a) Grasping and Releasing Objects**<br>• Difficulty holding objects due to **spasticity** (stiff muscles) or **weakness**.<br>• Trouble releasing objects voluntarily, sometimes dropping things unintentionally.<br>• Limited ability to perform precise grips (e.g., pincer grasp with thumb and index finger).<br>**b) Fine Motor Control**<br>• Difficulty with **hand–eye coordination** tasks such as stacking blocks, buttoning, or using utensils.<br>• Slow or clumsy movements when performing tasks requiring precision.<br>• Trouble with **bilateral coordination**, meaning using both hands together efficiently.<br>**c) Hand Posture and Position**<br>• Hands may be **stiff, curled, or open in abnormal positions** due to spasticity.<br>• Some children may have **floppiness** (hypotonia), making it hard to hold objects.<br>• Postural control issues may affect the ability to bring the hand to the mouth or face.<br>**d) Manipulation Skills**<br>• Difficulty **rotating, twisting, or moving objects** in the hand.<br>• Challenges with tasks like turning a pencil, unscrewing a lid, or manipulating toys.<br>**2. Underlying Causes**<br>Hand difficulties in CP are linked to:<br>• **Spasticity** (most common in spastic CP): Muscles are tight, limiting smooth movement.<br>• **Ataxia** (in ataxic CP): Poor coordination and tremor affect precision.<br>• **Dyskinesia/Athetosis**: Involuntary, uncontrolled movements interfere with deliberate hand use.<br>• **Muscle weakness**: Limits force generation needed for grip and manipulation.<br>• **Sensory impairments**: Some children have difficulty feeling objects, which impacts control.<br>**3. Functional Implications**<br>• Difficulty with **self-care tasks**: feeding, dressing, grooming.<br>• Problems with **school tasks**: writing, cutting, using scissors, typing.<br>• Limited participation in **play and sports** that require hand use.<br>• May lead to **frustration and reduced independence**.<br>**4. Support Strategies**<br>• **Occupational therapy (OT)**: Focused on improving hand function, grasp, and coordination.<br>• **Adaptive equipment**: Special utensils, writing aids, or splints to support function.<br>• **Home exercises and play-based activities**: Encourage bilateral hand use and strengthen fine motor skills.<br>• **Positioning and stretching**: To manage spasticity and improve range of motion.

**15-A5**	**Is it possible to improve my child's gross and fine motor skills if they have cerebral palsy?**	Yes—**it is absolutely possible to improve both gross and fine motor skills in a child with cerebral palsy (CP)**, although the extent of improvement depends on the type and severity of CP, the child's age, and the interventions used. The goal is usually **maximizing function, independence, and participation** rather than "curing" CP, since it is a lifelong condition.<br>**1. Gross Motor Skills**<br>These involve large muscle movements, like sitting, standing, walking, and balance.<br>**Ways gross motor skills can improve:**<br>• **Physical therapy (PT):**<br>o Exercises to strengthen muscles, improve balance, and increase range of motion.<br>o Activities to improve walking, crawling, or transfers (e.g., floor-to-stand).<br>• **Gait training and mobility aids:**<br>o Use of walkers, braces, or orthotics to support walking.<br>o Treadmill or supported walking programs can enhance strength and coordination.<br>• **Constraint-induced movement therapy (CIMT):**<br>o For children with hemiplegic CP, restraining the less affected side encourages the use of the weaker side.<br>• **Play-based therapy:**<br>o Structured play activities (climbing, ball games, obstacle courses) develop coordination and postural control.<br>**2. Fine Motor Skills**<br>These involve small movements, especially of the hands and fingers.<br>**Ways fine motor skills can improve:**<br>• **Occupational therapy (OT):**<br>o Exercises to strengthen hand muscles and improve coordination.<br>o Activities to develop grasp, release, and manipulation skills.<br>• **Task-specific practice:**<br>o Practicing self-care activities (buttoning, feeding) and school-related tasks (writing, using scissors).<br>• **Adaptive tools and splints:**<br>o Special utensils, pencil grips, or splints can help children perform tasks more independently while training their muscles.<br>• **Sensory-motor exercises:**<br>o Using clay, beads, or therapy putty to improve tactile feedback and finger strength.
**16-A2**	**What exercises can my child with cerebral palsy perform while using a wheelchair?**	Children with cerebral palsy (CP) who use wheelchairs can still do a variety of exercises to improve strength, flexibility, endurance, and overall well-being. The key is **safety, personalization, and enjoyment**. Here's a detailed breakdown:<br>**1. Upper Body Strengthening**<br>These exercises focus on the arms, shoulders, and chest, which are essential for mobility and independence:<br>• **Seated arm raises**: Lift arms to the side or overhead, with or without light weights.<br>• **Bicep curls**: Using resistance bands or light dumbbells.<br>• **Push-ups in wheelchair**: If safe, the child can push up from the chair armrests to lift their body slightly.<br>• **Wheelchair propulsion drills**: Practice short bursts or patterns to strengthen shoulder muscles.<br>**2. Core Strengthening**<br>A strong core supports posture, balance, and transfers:<br>• **Seated marches**: Lift knees alternately while seated.<br>• **Torso twists**: Rotate the upper body side to side slowly.<br>• **Forward and side bends**: Stretch and strengthen oblique and abdominal muscles.<br>• **Resistance band pulls**: Secure bands in front and pull outward to engage back and core muscles.<br>**3. Flexibility and Stretching**<br>Stretching helps reduce spasticity and maintain joint range of motion:<br>• **Neck stretches**: Gentle turns and tilts.<br>• **Shoulder stretches**: Arm across chest, overhead reach.<br>• **Wrist and finger stretches**: Important for fine motor function.

		• **Seated hamstring or calf stretches**: Use a strap or towel to extend legs if possible.<br>**4. Cardiovascular / Endurance**<br>Even in a wheelchair, aerobic exercise is possible:<br>• **Wheelchair sports**: Basketball, tennis, or racing in a safe space.<br>• **Seated dance or rhythm exercises**: Moving arms to music.<br>• **Arm cycling**: Using an upper-body ergometer.<br>• **Wheelchair intervals**: Alternating fast and slow pushing in a safe area.<br>**5. Functional and Daily Movement**<br>Exercises that improve independence and daily function:<br>• **Transfers practice**: Moving from wheelchair to bed, couch, or mat safely.<br>• **Reaching games**: Stretching for objects placed slightly out of reach to improve range of motion.<br>• **Grip and hand exercises**: Squeezing therapy putty, manipulating small objects.
**17-A3**	**What exercises should be done after calf muscle lengthening surgery for my child with cerebral palsy?**	After **calf muscle lengthening surgery** in a child with cerebral palsy (CP), the goal of post-operative exercises is to **maintain the new muscle length, regain strength, prevent contractures, and improve walking or mobility**. The exact program depends on the type of surgery (e.g., Achilles tendon lengthening) and the surgeon's protocol, but here's a structured overview:<br>**1. Early Phase (Immediately Post-Surgery – Usually 0–6 Weeks)**<br>**Goals:** Protect the surgical site, manage swelling, prevent stiffness, maintain circulation.<br>**Typical activities:**<br>• **Positioning & splints**: The ankle may be in a cast, splint, or boot to maintain the foot in proper alignment.<br>• **Gentle passive range-of-motion (ROM)**: Only if allowed by the surgeon, moving the ankle slowly within the allowed range.<br>• **Toe and knee movements**: While the leg is immobilized, moving other joints prevents stiffness and improves circulation.<br>• **Upper body and core exercises**: Seated exercises, wheelchair propulsion, or light arm strengthening to stay active.<br>⚠️ **Important:** Do **not** put weight on the operated leg until cleared by the surgeon or therapist.<br>**2. Intermediate Phase (After Cast/Splint Removal – Usually 6–12 Weeks)**<br>**Goals:** Gradually regain ankle ROM, improve muscle control, and start weight-bearing.<br>**Exercises:**<br>• **Gentle active-assisted ankle ROM**: The child gently moves the ankle within pain-free limits.<br>• **Stretching of the calf**: Hold for 20–30 seconds, multiple times per day. Start gently and increase as tolerated.<br>• **Strengthening exercises**:<br>○ **Towel scrunches**: Using toes to scrunch a towel while seated.<br>○ **TheraBand plantarflexion and dorsiflexion**: Light resistance to strengthen calf and shin muscles.<br>○ **Seated heel raises**: If allowed.<br>• **Balance and proprioception**: Gentle weight-shifting exercises if partial weight-bearing is allowed.<br>**3. Late Phase (3–6 Months Post-Surgery)**<br>**Goals:** Build strength, endurance, flexibility, and functional mobility.<br>**Exercises:**<br>• **Standing heel raises**: Start with support, progress to unassisted.<br>• **Mini squats and step-ups**: Focus on controlled motion.<br>• **Gait training**: Walking with orthoses or assistive devices as prescribed.<br>• **Functional activities**: Stair climbing, hopping, or playground activities to reinforce proper mechanics.<br>**Important Tips**<br>1. Always follow **surgeon and physical therapist instructions**, as timing and intensity vary.<br>2. **Pain or swelling** after exercise should be minimal; if it's severe, stop and consult the care team.<br>3. Use **fun, child-friendly approaches** (games, music, balance challenges) to improve participation.<br>4. Encourage **full participation in daily activities** once safe—walking, play, wheelchair propulsion—to prevent deconditioning.

**18-A3**	**What should I pay attention to after muscle lengthening surgery in my child with cerebral palsy?**	**Postoperative Considerations After Muscle Lengthening Surgery in Children with Cerebral Palsy**<br>1. **Pain Management:** Administer prescribed analgesics; monitor for uncontrolled pain.<br>2. **Wound Care:** Keep incisions clean and dry; watch for redness, swelling, discharge, or fever.<br>3. **Circulation and Swelling:** Elevate limb if advised; check skin color, warmth, and sensation.<br>4. **Immobilization:** Maintain casts, splints, or braces as instructed; avoid premature removal.<br>5. **Physical Therapy:** Initiate as prescribed; avoid overstretching or early weight-bearing.<br>6. **Spasticity Management:** Monitor changes in tone; adjust medications if needed.<br>7. **Complications:** Observe for infection, bleeding, neurovascular compromise, or cast-related issues.<br>8. **Nutrition and Support:** Ensure adequate protein and hydration; provide emotional support.<br>**Summary:** Careful monitoring, adherence to rehabilitation, and multidisciplinary coordination are essential for optimal recovery.
**19-A2**	**Are there home-based activities that my child with cerebral palsy can safely perform?**	There are many **safe, home-based activities** for children with cerebral palsy (CP) that can support mobility, strength, and coordination. The key is to **adapt activities to your child's abilities** and ensure safety<br>**1. Stretching and Range-of-Motion Exercises**<br>• Gentle, guided stretches for **hamstrings, calves, hips, and arms**.<br>• Can be done lying down, sitting, or supported standing.<br>• Helps maintain flexibility, prevent contractures, and improve posture.<br>**2. Strengthening Exercises**<br>• **Bodyweight activities:** Sit-to-stand from a chair, supported squats, wall push-ups.<br>• **Therapy bands:** Light resistance bands for arms and legs.<br>• Focus on **functional strength** (things your child uses daily).<br>**3. Balance and Coordination**<br>• Standing on a soft mat with support, shifting weight side to side.<br>• Sitting on a therapy ball (with supervision) to improve core stability.<br>• Simple stepping or obstacle courses in safe spaces.<br>**4. Functional Mobility Practice**<br>• Walking practice with or without assistive devices.<br>• Crawling or creeping activities for younger children.<br>• Stair practice if safe and supervised.<br>**5. Play-Based Activities**<br>• Dancing to music or moving with songs.<br>• Throwing/catching soft balls to improve coordination.<br>• Riding adapted tricycles or balance bikes (if allowed).<br>**6. Aquatic or Water Activities *(if accessible)***<br>• Swimming or water play can reduce stress on joints while promoting movement and strength.<br>**7. Fine Motor Skills**<br>• Beading, puzzles, drawing, or stacking blocks.<br>• Improves hand-eye coordination, dexterity, and grasp.
**20-A5**	**How can reduced grip strength be improved in my child with cerebral palsy?**	Reduced grip strength is common in children with cerebral palsy (CP) due to **spasticity, muscle weakness, or poor hand coordination**. The goal is to improve **functional hand use** safely and gradually. Here's a structured approach:<br>**1. Strengthening Exercises**<br>• **Therapy putty or clay:** Squeezing, pinching, or rolling shapes.<br>• **Stress balls:** Gentle squeezing and releasing.<br>• **Grip trainers:** Small hand grippers or spring-loaded devices (low resistance).<br>• **Finger extensions:** Rubber bands around fingers to open and close the hand.<br>**2. Functional Practice**<br>• **Daily tasks:** Turning doorknobs, zipping, buttoning, holding utensils, using pens.<br>• **Play-based tasks:** Building with blocks, stacking cups, playing with LEGO, or using toys requiring grasp and release.

		• **Adaptive tools:** Special grips or handles can make tasks easier and encourage practice.<br>**3. Fine Motor Coordination**<br>• Picking up small objects (coins, beads, buttons) to improve dexterity.<br>• Tracing, drawing, or cutting with child-safe scissors.<br>• Manipulating fasteners like Velcro or snaps to strengthen fingers.<br>**4. Stretching and Spasticity Management**<br>• Stretch fingers and wrists gently to reduce stiffness.<br>• Use **night splints** if recommended by a therapist to maintain finger extension.<br>• Regular **positioning and hand placement exercises** reduce contractures.<br>**5. Occupational or Physical Therapy**<br>• A therapist can create a **customized hand exercise program**, including electrical stimulation or constraint-induced movement therapy if appropriate.<br>• Therapy ensures exercises are safe, progressive, and target functional improvements.<br>**6. Encouragement and Motivation**<br>• Make exercises **fun and game-like**.<br>• Short, frequent practice sessions (5–10 min, multiple times a day) are more effective than long sessions.<br>**Summary:** Grip strength improves with **consistent practice, strengthening, fine motor activities, and spasticity management**. Collaboration with an occupational therapist ensures exercises are safe, age-appropriate, and goal-oriented.
**21-A2**	**How should I adjust exercise intensity for my child with cerebral palsy, and how many days per week should they exercise?**	Supporting a child with cerebral palsy (CP) through exercise requires careful consideration of **safety, goals, and individualized needs**. Here's a structured way to approach it:<br>**1. Adjusting Exercise Intensity**<br>Intensity depends on the type of activity, your child's abilities, and their Gross Motor Function Classification System (GMFCS) level. General principles:<br>**a. Aerobic/Cardio Exercise**<br>• **Goal:** Improve heart and lung fitness without overfatiguing muscles.<br>• **Intensity Guidance:**<br>o Use the **"talk test"**: Your child should be able to talk, but not sing, during activity.<br>o For some children, **moderate intensity** may be brisk walking, cycling, swimming, or adapted sports.<br>o Avoid high-intensity exercise that causes pain or excessive fatigue.<br>• **Duration:** Start with **10–20 minutes per session** and gradually increase to 20–30 minutes as tolerated.<br>**b. Strength/Resistance Training**<br>• **Goal:** Improve muscle strength, posture, and functional mobility.<br>• **Intensity Guidance:**<br>o Use **low to moderate loads**, focusing on form.<br>o **Repetitions:** 8–12 per set, 1–2 sets initially.<br>o Gradually increase resistance as tolerated.<br>o Avoid movements that strain joints or spastic muscles.<br>**c. Flexibility & Stretching**<br>• **Goal:** Maintain range of motion and prevent contractures.<br>• **Intensity Guidance:**<br>o Gentle, static stretching without bouncing.<br>o Hold stretches **15–30 seconds** per muscle group.<br>o Avoid overstretching spastic muscles, which can cause pain or injury.<br>**d. Balance & Coordination**<br>• Activities like standing on balance boards, gentle yoga, or adapted dance.<br>• Focus on **quality of movement**, not speed or intensity.<br>**2. Frequency of Exercise**<br>• **Aerobic activity:** Ideally **3–5 days per week**, starting lower and building tolerance.<br>• **Strength training: 2–3 non-consecutive days per week**.<br>• **Stretching: Daily**, especially for muscles prone to tightness (hamstrings, calves, hip flexors).<br>• **Balance/coordination practice:** Can be **daily** in short sessions (5–10 minutes).
**22-A5**	**Are there home-based**	**Home Hand Exercises for a Child with Cerebral Palsy**<br>**1. Warm-Up**

	**hand exercises I can do with my child who has cerebral palsy?**	• Shake hands, rotate wrists, tap fingers to thumb.<br>**2. Strengthening**<br>• **Squeeze ball/putty:** Hold 3–5 sec, 10–15 reps per hand.<br>• **Pinch objects:** Pick up coins/buttons, 5–10 reps.<br>• **Finger lifts:** Lift each finger from a flat hand, 5–10 reps.<br>**3. Flexibility & Range-of-Motion**<br>• **Wrist flex/extend:** 10–15 reps.<br>• **Finger stretch:** Spread and close fingers, 10–15 reps.<br>**4. Coordination & Functional Skills**<br>• Buttoning, zipping, picking up small objects, play dough activities.<br>**5. Frequency**<br>• **Daily**, 10–15 min. Short, fun sessions work best.<br>**Tip:** Keep it playful, use both hands, stop if painful.
**23-A1**	**Can cerebral palsy be completely cured with treatment?**	No, **cerebral palsy (CP) cannot be completely cured**. CP is caused by **brain damage or abnormal development** that occurs before, during, or shortly after birth, and that brain injury is **permanent**.<br>However, **treatment can significantly improve function, independence, and quality of life**:<br>**Goals of Treatment**<br>• **Improve movement and coordination**: Physical and occupational therapy, stretching, strengthening exercises.<br>• **Reduce complications**: Manage spasticity, contractures, or joint deformities.<br>• **Support daily activities**: Assistive devices (braces, walkers), adaptive equipment.<br>• **Manage associated conditions**: Speech, vision, hearing, or cognitive challenges.<br>• **Enhance participation**: Sports, play, and social activities.<br>**Treatment Approaches**<br>1. **Therapies**<br>o Physical therapy for mobility and strength.<br>o Occupational therapy for daily activities.<br>o Speech therapy for communication and swallowing.<br>2. **Medications**<br>o To reduce muscle spasticity or seizures.<br>3. **Surgery**<br>o Orthopedic or neurosurgical procedures in select cases.<br>4. **Assistive devices**<br>o Walkers, wheelchairs, braces, communication aids.
**24-A2**	**Does exercise reduce spasticity in children with cerebral palsy?**	**Yes**, exercise can help reduce or manage spasticity in children with cerebral palsy (CP), though the effects depend on the type of exercise, how it's done, and the individual child. Here's a breakdown of what the research says, plus some caveats.<br>**What the Evidence Shows**<br>1. **Strength Training**<br>o A systematic review and meta-analysis found that strength (resistance) training in children and adolescents with *spastic* CP improved muscle strength, balance, gait speed, and gross motor function **without increasing spasticity**.<br>o So, strength training doesn't necessarily worsen spasticity and may actually support better motor function.<br>2. **Aquatic Exercise**<br>o Studies compared aquatic (water) exercise to land-based exercise in children with CP. Both groups improved in functional measures, but the aquatic group showed **better improvements in quality of life**.<br>o Using ultrasonography, researchers also saw better "muscle compressibility" (i.e., more elastic muscle) in the aquatic group, suggesting a physiological change in muscle.<br>o The authors note that warm water immersion may reduce gamma-fiber activity (part of the stretch reflex), helping with muscle relaxation and spasticity.<br>3. **Regular Physical Therapy (Stretching / Flexible Exercise)**<br>o In a review of physical therapy in children with CP, researchers found that **regular therapy** (more than just occasional therapy) helps maintain range of motion (ROM) and prevent deterioration in spastic joints.

		○ Specifically, stretching 1–3 times a week over at least 8 weeks was shown to *decrease spasticity* in some studies.<br>○ The mechanisms may be both **neuroplastic** (i.e., changing reflex sensitivity) and **mechanical** (changing muscle properties like length or stiffness).<br>4. **Home-Based Programs & Massage**<br>○ A more recent randomized trial tested a home-based exercise program (delivered by caregivers) plus pediatric massage. After **6 and 12 weeks**, kids receiving massage + exercises had a **statistically significant drop in spasticity**, compared to exercise alone.<br>○ This suggests that combining modalities (exercise + massage) might be more effective for tone management.<br>5. **Higher-Level Evidence (Umbrella Review)**<br>○ An umbrella review (i.e., a review of systematic reviews) showed that physical activity/exercise (particularly *task-oriented* training and VR-assisted training) consistently improves motor and limb function in CP.<br>○ However, while these reviews document many positive effects, **direct and consistent evidence for spasticity reduction is more limited** (i.e., not all forms of exercise reduce spasticity, and not all outcomes are about tone).
**25-A1**	**Does cerebral palsy worsen over time?**	Cerebral palsy (CP) itself **does not worsen over time**, because it is caused by a **non-progressive brain injury** that occurs during brain development, usually before, during, or shortly after birth. The brain damage that leads to CP does not continue to deteriorate.<br>However, there are some nuances to be aware of:<br>1. **Secondary complications may appear or worsen**<br>○ Muscle stiffness (spasticity), joint contractures, or scoliosis can develop or progress as a person grows.<br>○ Pain, fatigue, and mobility challenges may increase over time due to these secondary musculoskeletal issues.<br>2. **Functional abilities may change with age**<br>○ Children may "outgrow" their motor skills if therapy isn't consistent.<br>○ Adults with CP sometimes experience earlier onset of musculoskeletal problems like arthritis, which can make movement more difficult.<br>3. **Some forms can seem to worsen in appearance**<br>○ Spasticity or abnormal gait may become more noticeable with growth, but this is due to **body changes** rather than the underlying brain injury worsening.
**26-A5**	**How can bimanual (two-handed) activities be improved in my child with cerebral palsy?**	Improving **bimanual (two-handed) activities** in a child with cerebral palsy (CP) usually focuses on **coordination, strength, and functional use of both hands together**. Here's a detailed, practical approach:<br>**1. Task-Specific Practice**<br>Focus on **meaningful activities your child enjoys**, because motivation drives improvement. Examples:<br>• Buttoning shirts<br>• Opening containers<br>• Cutting with scissors<br>• Building with blocks<br>• Using both hands in play (throwing/catching, stacking, drawing)<br>**Tip:** Break tasks into smaller steps and practice the most challenging parts repeatedly.<br>**2. Constraint-Induced Movement Therapy (CIMT)**<br>• This is often used for children with hemiplegic CP (one side affected).<br>• The **stronger hand is gently restrained** for short periods to encourage use of the weaker hand, then integrate both hands in tasks.<br>• Can be done at home with supervision, under guidance from a therapist.<br>**3. Bimanual Training Programs**<br>• Practice tasks **requiring both hands to work together**, emphasizing coordination rather than just strength.<br>• Examples:<br>○ Opening a jar (one hand stabilizes, one hand turns)<br>○ Folding towels<br>○ Playing musical instruments (drums, piano)<br>**4. Strengthening & Stretching**<br>• **Target weak muscles** (grip, forearm, wrist, shoulder).

		• Use **theraputty, hand exercisers, or small weights**.<br>• Stretch tight muscles to prevent spasticity from limiting movement.<br>**5. Fine Motor Skills Practice**<br>• Activities that improve dexterity also help bimanual tasks:<br>○ Picking up small objects (coins, beads)<br>○ Threading beads or laces<br>○ Playing with Lego or puzzles<br>**6. Adaptive Tools & Supports**<br>• Special utensils, cups, scissors, and handles can help your child participate successfully, which **encourages practice** and reduces frustration.<br>**7. Therapy Guidance**<br>• **Occupational therapists (OTs)** are specialists in bimanual skills.<br>• They can create **personalized home programs**, teach proper positioning, and introduce fun activities that strengthen both hands togethe
**27-A1**	**What does spasticity mean?**	**Spasticity** is a condition in which certain muscles are continuously tight or stiff, making movement difficult. It happens because the **brain or spinal cord signals that control muscle activity are disrupted**, as often occurs in cerebral palsy.<br>Key points:<br>• Muscles resist stretching and may feel "hard" or tense.<br>• Movements can be jerky, stiff, or awkward.<br>• Severity can range from mild tightness to muscles that are very rigid.<br>• Spasticity can affect posture, walking, and the ability to perform daily tasks.<br>**Tip:** Spasticity is not the same as muscle weakness—it's an **excessive muscle contraction** caused by abnormal nerve signals. Therapy, stretching, medication, or sometimes surgery can help manage it.
**28-A3**	**I have a child with cerebral palsy. What is botulinum toxin (Botox), and does it reduce spasticity?**	Children with CP often have **spasticity**, which is increased muscle tone that can cause stiffness, tightness, or involuntary muscle contractions. Botox is injected directly into specific spastic muscles. By reducing the muscle's overactivity, it can:<br>• **Decrease spasticity** (make the muscle less stiff)<br>• **Improve range of motion**<br>• **Make stretching, therapy, or splinting easier**<br>• Sometimes, it can also **reduce pain or improve gait** if used in leg muscles<br>**Who might benefit:**<br>• Children with **focal spasticity** (specific muscles, not generalized)<br>• Those who need improved function for therapy or daily activities<br>• Sometimes before orthopedic surgery to improve outcomes<br>In short, **yes, Botox can reduce spasticity** in children with CP, especially when targeted to specific muscles, but it works best as part of a comprehensive treatment plan.
**29-A5**	**Is restricting the more frequently used hand beneficial for encouraging the use of the less-used hand in my child with cerebral palsy?**	Yes, restricting the more frequently used hand **can be beneficial** in certain cases for children with cerebral palsy, but it must be done carefully and under professional guidance. What you're describing is known as **Constraint-Induced Movement Therapy (CIMT)**, a well-studied intervention in pediatric rehabilitation. Here's a detailed breakdown:<br>**How CIMT Works**<br>• The more functional hand (typically the dominant or less affected hand) is **temporarily restrained** using a mitt, splint, or sling.<br>• The child is then **encouraged or guided** to use the weaker or less-used hand during daily activities and structured therapy tasks.<br>• This helps the brain **reorganize motor pathways** and reduces "learned non-use" of the affected limb.<br>**Benefits**<br>• Improved **strength, dexterity, and coordination** in the less-used hand.<br>• Increased **functional use** of the weaker hand in everyday tasks.<br>• Can be particularly effective in children with **unilateral cerebral palsy** (one side affected).<br>**Considerations & Cautions**<br>1. **Age & Development:** CIMT is typically used in children aged **2–12 years**, but younger or older children can sometimes benefit.<br>2. **Intensity & Duration:** Usually involves **a few hours a day to most of the day for several weeks**, but exact schedules are individualized.<br>3. **Supervision:** Must be **guided by an occupational or physical therapist** to ensure safety, proper technique, and avoid frustration or injury.

		4. **Child's Tolerance:** Some children resist restraint; therapy should remain **motivating and playful**.<br>5. **Not Always Suitable:** Children with severe spasticity, contractures, or other medical issues may require alternative approaches.
**30-A1**	**Does cerebral palsy affect my child's vision and hearing?**	Cerebral palsy (CP) primarily affects movement and muscle tone, but it can also be associated with other developmental issues, including vision and hearing problems. However, the effects vary widely depending on the type and severity of CP and whether there are additional neurological or sensory complications.<br>**Vision**<br>Children with CP may experience a range of visual problems, such as:<br>• **Strabismus (crossed eyes)**: Misalignment of the eyes.<br>• **Refractive errors**: Nearsightedness, farsightedness, or astigmatism.<br>• **Cortical visual impairment (CVI)**: The brain has difficulty processing visual information even though the eyes themselves may be healthy.<br>• **Nystagmus**: Involuntary eye movements.<br>• **Difficulty with depth perception or tracking moving objects**.<br>The likelihood of visual problems is higher in children with more severe CP or those born prematurely. Regular eye exams are important.<br>**Hearing**<br>Hearing problems are less common than vision problems but can still occur:<br>• **Sensorineural hearing loss**: Often due to damage to the inner ear or auditory nerve, sometimes linked to prematurity or perinatal complications.<br>• **Conductive hearing loss**: May result from frequent ear infections.<br>• **Auditory processing issues**: The brain may have difficulty interpreting sounds even if hearing is normal.<br>Early hearing screening is important to detect any issues, as untreated hearing problems can affect speech and language development.
**31-A2**	**How does exercise affect the mental health of my child with cerebral palsy?**	Exercise can have a significant positive impact on the mental health of a child with cerebral palsy (CP), in ways that go beyond just improving physical strength or mobility. Here's a detailed breakdown:<br>**1. Mood Improvement**<br>• **Endorphin release:** Physical activity stimulates the release of endorphins and other neurotransmitters (like serotonin and dopamine), which naturally improve mood and reduce feelings of anxiety or sadness.<br>• **Stress reduction:** Exercise can help your child cope with the stress and frustration that sometimes accompany physical limitations.<br>**2. Reduced Anxiety and Depression**<br>• Children with CP are at higher risk for anxiety and depression due to social challenges, physical limitations, and frequent medical interventions.<br>• Regular exercise—even adapted forms like swimming, yoga, or physiotherapy-based movement—can lower symptoms of anxiety and depression by providing a structured, achievable activity that promotes a sense of control and accomplishment.<br>**3. Improved Self-Esteem and Confidence**<br>• Mastering new skills or participating in group activities can boost self-esteem.<br>• Even small improvements in strength, coordination, or stamina can make daily tasks easier, enhancing independence and confidence.<br>**4. Social Interaction**<br>• Group exercise or adaptive sports programs provide opportunities for social engagement, teamwork, and friendship, which are crucial for emotional well-being.<br>• Feeling part of a team or community can reduce feelings of isolation that some children with CP experience.
**32-A4**	**Can I help improve my child's walking speed?**	Helping a child improve walking speed is possible, but it's important to approach it safely, making sure it's age-appropriate and supports healthy development. Here's a structured approach:<br>**1. Assess the baseline**<br>• Observe your child walking: Are they hesitant, dragging feet, or just naturally slow?<br>• Note if there's any pain, fatigue, or imbalance—these may indicate an underlying issue that should be checked by a pediatrician or physical therapist.<br>**2. Encourage general leg strength**<br>Stronger legs improve walking speed and stability. Try:<br>• **Squats or mini-squats**: Can be done holding onto a chair for support.

		• **Step-ups**: Using a safe step, have your child step up and down repeatedly.<br>• **Lunges or walking lunges**: If they're old enough and stable.<br>• **Jumping games**: Hopscotch, jumping jacks, or trampoline play.<br>**3. Practice walking itself**<br>• **Short "speed bursts"**: Encourage walking a bit faster for 10–20 seconds, then normal pace.<br>• **Obstacle courses**: Place cones or toys to navigate quickly but safely.<br>• **Walking on different surfaces**: Grass, sand, or mats can improve balance and agility.<br>**4. Work on posture and gait**<br>• Encourage **upright posture** with shoulders back and relaxed.<br>• Teach **arm swing coordination** with steps—it naturally increases pace.<br>• Focus on **stride length**: Practicing longer steps without running can help speed.<br>**5. Games and motivation**<br>Kids respond well to play:<br>• "Race the clock" for short distances.<br>• Follow-the-leader games where the leader varies speed.<br>• Treasure hunts where they need to walk faster to find items.
**33-A4**	**Why does my child with cerebral palsy experience knee hyperextension while walking?**	Knee hyperextension (also called **genu recurvatum**) during walking is a common issue in children with **cerebral palsy (CP)**, especially those with **spasticity**. The reason your child experiences it involves a combination of **muscle imbalance, altered joint mechanics, and compensatory strategies**. Let's break it down carefully.<br>**1. Muscle Imbalance**<br>• In CP, certain muscles are **spastic** (overactive) and others are **weak**.<br>• **Quadriceps weakness**: The quadriceps normally control knee flexion during the stance phase. If weak, the knee may snap backward.<br>• **Plantarflexor spasticity (calf muscles)**: Tight or spastic calf muscles (gastrocnemius and soleus) push the ankle into plantarflexion. This can **force the knee into hyperextension** as the leg tries to stabilize during weight-bearing.<br>**2. Altered Gait Mechanics**<br>• During **stance phase**, the knee should be slightly flexed to absorb shock.<br>• If the child has **spasticity or weakness** in key muscles:<br>○ The knee may **snap backward** to maintain stability.<br>○ This is sometimes a **compensation for poor hip or ankle control**.<br>• Essentially, the body "locks" the knee to prevent falling forward when muscle control is insufficient.<br>**3. Joint and Soft Tissue Factors**<br>• **Ligamentous laxity**: Some children with CP have looser ligaments, making hyperextension easier.<br>• **Reduced ankle dorsiflexion**: If the ankle can't bend upward properly, the knee bends backward to keep the foot on the ground.<br>**4. Compensation for Weakness**<br>• If the **hip extensors or gluteal muscles** are weak, the child may **push the knee backward** to help keep the center of mass over the foot.<br>• Sometimes this is called a **"stiff-legged" or "extension" strategy**.
**34-A1**	**How does cerebral palsy affect bones and joints?**	Cerebral palsy (CP) can significantly affect bones and joints due to abnormal muscle tone, spasticity, and uneven forces on the skeleton. Muscles that are tight or weak can pull unevenly on bones, leading to deformities such as scoliosis, hip dislocation, or foot and knee misalignments. Reduced mobility and limited weight-bearing can cause bones to grow abnormally or become less dense, increasing the risk of fractures. Joints may become stiff or develop contractures, restricting range of motion, while abnormal gait patterns can accelerate wear and tear, potentially causing pain and early arthritis. Over time, these musculoskeletal changes can affect posture, mobility, and overall function.
**35-A3**	**At what age can muscle lengthening surgery be performed for calf muscles in children with cerebral palsy?**	There's not a **single** universally agreed "minimum age," but expert consensus and research give us good guidance for calf (gastroc-soleus) muscle lengthening in children with cerebral palsy (CP):<br>**What the Evidence & Expert Guidelines Say**<br>1. **Delphi consensus study:**<br>○ A panel of experienced pediatric orthopedic surgeons reached *general agreement* that the **optimum age** for gastrocsoleus lengthening is between **6 and 10 years**.<br>○ They also agreed that surgery should **be avoided in children under 4 years**.

		○ Zone-3 (i.e., Achilles tendon) lengthening was considered contraindicated in some subtypes.<br>2. **Clinical practice data:**<br>○ In one study of isolated calf lengthening, the average age at surgery was **8.8 years** (range 6–14).<br>○ Complication risk: In that same study, children aged **8 years or younger** had a **much higher risk of calcaneus deformity** (i.e., over-lengthening) than those older than eight.<br>○ According to a pediatric orthopedics textbook: delaying surgery until **6–8 years** helps reduce risk of over-lengthening and secondary gait problems like crouch gait.<br>3. **Other practical guidelines:**<br>○ Some clinical-practice guidelines note that most very young children (e.g., under **4–6 years**) do **not require** such surgery, unless there are fixed deformities.<br>○ According to a therapy manual, some recommend **waiting until around 8 years** before Achilles lengthening to reduce the need for repeat surgery later.
**36-A3**	**When should I begin stretching exercises after my child with cerebral palsy receives botulinum toxin?**	The timing of stretching exercises after botulinum toxin (Botox) injections for children with cerebral palsy is important to maximize benefit and minimize risks.<br>**1. Understanding the Purpose**<br>Botulinum toxin temporarily weakens spastic muscles by blocking nerve signals. Stretching and positioning after the injection help maintain **range of motion (ROM)** and take advantage of the period when the muscle is more relaxed.<br>**2. Typical Timing**<br>• **Immediate Post-Injection (within 24–48 hours):**<br>○ Gentle, passive stretching can usually begin almost immediately, as the injection itself doesn't require waiting for effect.<br>○ The toxin generally takes **2–7 days** to start relaxing the muscle noticeably.<br>• **During the Peak Effect (1–4 weeks post-injection):**<br>○ Stretching and strengthening exercises are **most effective** during this window because the muscles are less resistant, making it easier to improve joint ROM.<br>• **Ongoing Maintenance:**<br>○ Stretching should continue regularly, as the effect of botulinum toxin is temporary (typically 3–6 months), and the goal is to prevent contractures.
**37-A2**	**Can my child with cerebral palsy participate in physical education classes at school?**	Most children with cerebral palsy **can participate in physical education (PE) classes**, but their participation may need to be **adapted** to their abilities and needs. The key is ensuring **safety, inclusion, and appropriate support**. Here's a detailed breakdown:<br>**1. Assessment First**<br>• A physical therapist, occupational therapist, or your child's doctor can evaluate **strength, balance, coordination, endurance, and mobility**.<br>• This assessment helps determine **which activities are safe** and which might need modifications.<br>**2. Individualized Adaptations**<br>• **Activity modifications:** For example, using a lighter ball, lowering nets, or allowing wheelchair-based versions of sports.<br>• **Equipment adaptations:** Cushioned mats, supportive braces, or adaptive bikes can help participation.<br>• **Pacing adjustments:** Allow more time for transitions, rest breaks, or fewer repetitions.<br>**3. Collaboration**<br>• Teachers, therapists, and parents should **communicate regularly**.<br>• Many schools can provide an **Individualized Education Program (IEP)** or **504 Plan** in the U.S., which can include **specific PE accommodations**.<br>**4. Social and Emotional Benefits**<br>• Participation can improve **strength, coordination, cardiovascular health**, and **self-esteem**.<br>• Inclusive PE fosters **friendships and teamwork**, which are just as important as physical fitness.

**38-A1**	**What can be done to reduce spasticity?**	Spasticity is a condition where certain muscles are continuously contracted, causing stiffness, tightness, and sometimes pain. It's often seen in conditions like **stroke, cerebral palsy, multiple sclerosis, or spinal cord injury**. Reducing spasticity typically involves a combination of **medical, physical, and sometimes surgical approaches**. Here's a detailed breakdown:<br>**1. Physical and Occupational Therapy**<br>• **Stretching exercises:** Regular, gentle stretching helps maintain muscle length and prevent contractures.<br>• **Range-of-motion exercises:** Moving joints through their full range can reduce stiffness.<br>• **Strengthening opposing muscles:** Strengthening weaker muscles can help balance muscle activity.<br>• **Positioning and splinting:** Splints, braces, or proper seating/lying positions can prevent muscles from shortening.<br>**2. Medications**<br>• **Oral medications:**<br>○ **Baclofen:** Reduces muscle tone by acting on the central nervous system.<br>○ **Tizanidine:** Short-acting muscle relaxant, reduces spasticity and muscle stiffness.<br>○ **Diazepam:** Sometimes used for spasticity with anxiety or sleep issues.<br>○ **Dantrolene:** Works directly on muscles, usually for severe generalized spasticity.<br>• **Botulinum toxin injections (Botox):**<br>○ Targeted at specific muscles.<br>○ Can reduce localized spasticity for 3–6 months.<br>• **Intrathecal Baclofen Pump:**<br>○ Delivers medication directly into the spinal fluid for severe or generalized spasticity.<br>**3. Surgical and Procedural Interventions**<br>• **Tendon release or lengthening:** For muscles that are severely tight.<br>• **Selective dorsal rhizotomy:** Cutting nerve fibers to reduce spasticity (usually for cerebral palsy).<br>• **Orthopedic surgery:** To correct deformities caused by long-term spasticity.
**39-A4**	**What exercises can I do to improve my child's balance?**	Improving a child's balance is all about **core strength, coordination, proprioception (body awareness), and motor planning**. The exercises depend on your child's age and abilities, but here's a structured approach that can be used safely at home or in therapy:<br>**1. Core and Trunk Strength**<br>Strong core muscles are crucial for balance. Try these:<br>• **Plank variations**:<br>○ Standard plank on elbows or hands.<br>○ Side plank for obliques.<br>○ Start with 5–10 seconds and gradually increase.<br>• **Superman exercise**: Lying on the stomach, lift arms and legs simultaneously.<br>• **Seated balance games**: Sit on a therapy ball or cushion and reach in different directions.<br>**2. Static Balance Exercises**<br>These focus on holding positions without movement:<br>• **Single-leg stands**:<br>○ Stand on one leg while holding a wall or chair.<br>○ Progress by letting go of support, then closing eyes.<br>• **Tightrope walk**: Walk heel-to-toe along a line or tape on the floor.<br>• **Balance board or wobble board**: Gentle tilting exercises help stabilize muscles.<br>**3. Dynamic Balance Exercises**<br>These involve movement while maintaining control:<br>• **Obstacle courses**: Step over cushions, crawl under a table, walk around cones.<br>• **Hopscotch**: Encourages coordination, leg strength, and balance.<br>• **Lunges and squats**: Improves lower body strength and stability.<br>**4. Coordination and Proprioception**<br>Exercises that improve body awareness:<br>• **Ball games**: Catching, throwing, or kicking a ball.

		• **Marching or hopping games**: Hop on one foot, march in place, or jump in patterns.<br>• **Yoga for kids**: Simple poses like tree pose, warrior, or downward dog improve both balance and flexibility.<br>**5. Functional and Everyday Practice**<br>Balance improves most with activities that mimic real life:<br>• Walking on curbs or uneven surfaces.<br>• Climbing stairs without holding the railing (if safe).<br>• Dancing or following movement-based games (like "Simon Says" or dance videos).
**40-A3**	**Is it safe for my child with cerebral palsy to perform exercises after receiving botulinum toxin injections?**	**Exercise after botulinum toxin injections can be safe and is often recommended**, but it must be done carefully and under professional guidance. Here's a detailed explanation:<br>**Why Exercise is Important After Botox**<br>• Botox **reduces muscle overactivity** (spasticity) temporarily.<br>• Exercises help **maintain range of motion, strengthen opposing muscles, and improve functional outcomes**.<br>• Without follow-up therapy, the benefits of Botox may be **short-lived**.<br>**Timing**<br>• **Immediate post-injection:** Usually, gentle stretching can begin **right after injection**.<br>• **Therapy progression:** Physical or occupational therapy is typically resumed **within 24–48 hours**, focusing on stretching, range-of-motion, and gradual strengthening.<br>• **Intense exercise:** High-impact or very strenuous exercises are usually avoided for the first few days if the child experiences soreness at injection sites.<br>**Recommended Exercises**<br>• **Gentle stretching** of injected muscles.<br>• **Active range-of-motion exercises** to encourage functional movement.<br>• **Balance and coordination activities** appropriate for your child's ability.<br>• **Functional activities** like walking, reaching, or play-based movements.
**41-A4**	**My child with cerebral palsy is able to walk. What type of footwear should I choose?**	Choosing the right footwear for a child with cerebral palsy (CP), even when they can walk, is really important — the right shoes can help with stability, comfort, and long-term foot development. Here are some considerations + what to look for, and a few product ideas. (Of course, it's also good to talk to your child's physiotherapist or orthotist — they know their specific needs.)<br>**What to Look for in Footwear**<br>1. **Support & Stability**<br>○ A firm **heel counter** (the back part of the shoe) helps with stability and prevents the shoe from collapsing.<br>○ A **wide or stable base** (wide sole) is helpful for balance.<br>○ If your child uses an **AFO or orthotic insoles**, you'll want shoes that can accommodate them.<br>2. **Adjustability**<br>○ Look for shoes with **Velcro straps, laces, or adaptive closures** — easy to adjust, secure fit, and can accommodate braces or swelling.<br>○ Wide openings (shoes that "open down toward the toes") make it easier to put shoes on, especially over braces.<br>3. **Sole & Cushioning**<br>○ Non-slip soles are safer, especially if there's balance issues or irregular gait.<br>○ Good shock absorption / cushioning helps reduce impact and protect joints.<br>○ A "rocker" or curved forefoot in the sole can help with smoother walking motion.<br>4. **Weight & Material**<br>○ Lightweight shoes reduce fatigue, making walking easier.<br>○ Breathable, comfortable materials help — especially if wearing braces or insoles.<br>5. **Fit & Size**<br>○ Make sure the shoes are the right length and width. Too small = pressure, too big = instability.<br>○ Removable insoles are very helpful: you can take them out if your child has orthotics.<br>6. **Easy On/Off**

		○ Shoes that are easy to put on/off help your child be more independent.<br>○ Some adaptive designs (like zip + laces) make wearing braces or orthotics easier.<br>7. **Avoid**<br>○ Avoid slip-on shoes that don't adjust well.<br>○ Avoid very flexible, flimsy shoes — they don't provide enough support.<br>○ High-heeled shoes; they're unstable and can put pressure in the wrong places.<br>8. **Orthotic Inserts**<br>○ Consider using insoles designed for CP (or more generally for stability) — e.g., bespoke or semi-custom orthotic insoles to support the arch and ankle.
**42-A3**	**Which exercises should be performed after serial casting is completed in my child with cerebral palsy?**	After **serial casting** in a child with cerebral palsy (CP), the goal is usually to **maintain the increased range of motion**, improve **muscle strength**, and encourage **functional mobility**. The exact exercises depend on the child's type of CP, affected muscles, and any residual contractures, but here's a comprehensive guide:<br>**1. Stretching / Range of Motion (ROM) Exercises**<br>The main goal is to **maintain the length gained from serial casting**.<br>• **Calf Stretch / Ankle Dorsiflexion Stretch**<br>○ Sit or lie with the leg straight.<br>○ Gently pull the toes toward the shin (ankle dorsiflexion).<br>○ Hold for **20–30 seconds**, repeat **3–5 times** per session, **2–3 times per day**.<br>○ Avoid bouncing; slow, sustained stretches are safest.<br>• **Hamstring & Quadriceps Stretch** (if lower limb affected)<br>○ Straight leg raise stretches hamstrings.<br>○ Gentle knee flexion stretches quadriceps.<br>• **Hip and Knee ROM**<br>○ Slowly flex and extend the hip and knee to maintain mobility.<br>**2. Strengthening Exercises**<br>After casting, muscles may be weaker due to immobilization. Focus on **antagonist and functional muscles**:<br>• **Ankle & Foot Strengthening**<br>○ **Heel raises**: Stand holding a support, lift heels up and down.<br>○ **Toe raises**: Strengthen tibialis anterior (dorsiflexor) for walking.<br>○ **Resistance band exercises**: Pull foot against band in dorsiflexion/plantarflexion directions.<br>• **Leg Strengthening**<br>○ **Bridging**: Lie on back, lift hips off floor to strengthen glutes.<br>○ **Mini-squats**: Hold support, bend knees slightly.<br>○ **Step-ups**: Step onto a low platform.<br>• **Core Stability**<br>○ Sit-to-stand practice.<br>○ Balance activities on a therapy ball or wobble board (if safe).<br>**3. Gait and Functional Training**<br>Once cleared by the physiotherapist, focus on **walking and weight-bearing activities**:<br>• **Treadmill walking** (with support if needed)<br>• **Overground walking** on different surfaces<br>• **Balance activities**: Standing on one leg (with support), stepping over obstacles.<br>**4. Splint or Orthotic Use**<br>• Children may be prescribed **AFOs (ankle-foot orthoses)** or **night splints** after casting.<br>• Continue to wear as directed to **maintain ankle dorsiflexion** and **prevent recurrence of contracture**.
**43-A2**	**What types of equipment should I use when exercising with my child who has cerebral palsy?**	Exercising with a child who has **cerebral palsy (CP)** requires careful attention to safety, support, and the child's individual abilities. The right equipment can help improve strength, balance, flexibility, coordination, and confidence while minimizing the risk of injury.<br>**1. Supportive and Safety Equipment**<br>• **Adaptive seating or supportive chairs:** For children with limited trunk control. These help them maintain posture during exercises or stretching.<br>• **Harnesses and suspension systems:** Used for gait training or assisted walking. They reduce fall risk and allow partial weight-bearing practice.

		• **Therapy mats and padded surfaces:** Provide cushioning for floor exercises, rolling, or stretching.<br>**2. Mobility Aids**<br>• **Walkers or gait trainers:** For children who can take steps but need balance support. Some are adjustable for height and resistance.<br>• **Canes or crutches:** If recommended by a physical therapist for children who can bear weight but need balance assistance.<br>• **Wheelchairs (manual or power):** Can be used for upper-body exercises if walking is not possible.<br>**3. Strength and Coordination Equipment**<br>• **Resistance bands:** Gentle, low-impact way to strengthen muscles. They can be used for arms, legs, and core exercises.<br>• **Light hand weights or medicine balls:** Only if the child can safely grasp and control them. Focus on functional strength.<br>• **Therapy balls (exercise or physioballs):** Great for balance, core strengthening, and flexibility.<br>• **Weighted vests or ankle/wrist weights:** Sometimes used under guidance of a therapist to improve postural control.<br>**4. Balance and Sensory Equipment**<br>• **Balance boards or wobble cushions:** Helps develop balance and proprioception. Should always be used with supervision.<br>• **Trampolines (mini or therapy trampoline):** Useful for low-impact strengthening and coordination.<br>• **Foam rollers or therapy rolls:** Good for stretching and gentle core exercises.<br>**5. Fun and Motivating Tools**<br>• **Obstacle courses or cones:** Encourage movement, coordination, and motor planning in a playful way.<br>• **Balls of different sizes and textures:** For catching, kicking, rolling—improves hand-eye coordination and gross motor skills.<br>• **Interactive games (video-based or apps):** Can encourage movement in a motivating, engaging way, especially for children with limited mobility.
**44-A5**	**What activity suggestions are appropriate for tasks performed with one hand in children with cerebral palsy?**	For children with cerebral palsy (CP) who primarily use **one hand due to hemiplegia or limited function on one side**, activities should focus on **strengthening the affected hand, improving coordination, and building independence**. The key is to make the activities **functional, fun, and repetitive** without causing frustration.<br>**1. Fine Motor Activities**<br>Activities that target **finger dexterity, grasp, and release**:<br>• **Buttoning and zipping practice:** Use shirts, jackets, or dressing boards.<br>• **Stacking and building:** Blocks, LEGO, or stacking cups.<br>• **Bead threading:** Large beads at first, then smaller ones as skill improves.<br>• **Playdough or clay exercises:** Rolling, pinching, or cutting strengthens fingers and hand muscles.<br>• **Scooping, pouring, or transferring small objects:** Using cups, spoons, or tongs.<br>**2. Gross Motor and Bilateral Coordination**<br>Even if one hand is weaker, activities should encourage **whole-arm engagement** and balance:<br>• **Pushing, pulling, or rolling balls:** Large balls rolled toward a target help coordination.<br>• **Pushing toy cars or trucks:** Encourages weight shift and arm movement.<br>• **Clapping or hitting a balloon:** Balloon activities are slow, fun, and encourage reaching.<br>• **Swinging on a swing or hanging from monkey bars:** Use one hand if safe, or alternate with assistance.<br>**3. Adaptive Self-Care Tasks**<br>These can be practiced as **functional one-handed skills**:<br>• **Eating with a spoon or fork** held in the more functional hand.<br>• **Brushing teeth or hair** using adaptive grips if needed.<br>• **Opening containers:** Use jars with rubber grips or adaptive openers.<br>**4. Hand Strengthening Tools**<br>• **Therapy putty or squeezable balls** to increase grip strength.<br>• **Clothespins or spring-loaded clamps** for pinching exercises.

		• **Velcro toys or fasteners** that require controlled pressing and pulling.<br>**5. Creative and Play-Based Activities**<br>• **Painting, stamping, or finger-painting** with one hand.<br>• **Music instruments:** Drums, tambourines, or keyboards for rhythm and coordination.<br>• **Interactive video games or tablet apps** that respond to single-hand input.<br>• **Sticker books or simple crafts** that encourage precise placement and manipulation.
**45-A4**	**What technological or game-based equipment can help improve walking and balance in my child with cerebral palsy?**	**Virtual reality-Augmented Treadmill Training**<br>• Combining treadmill walking + virtual reality improves gait speed, balance, and muscle strength in kids with CP.<br>• One study used Nintendo Wii + treadmill for this.<br>**Exergaming (Video-Games + Motion Sensors)**<br>• Home-based exergame (Xbox Kinect) training improved balance in children with spastic CP, especially those who had *low baseline balance*.<br>• Kinect v2 sensors have also been used for gait analysis in CP, because they're low cost and track body movement.<br>• Local RCT from Türkiye showed benefits of Kinect games on balance and motor function.<br>**Robotic / Gait-Assist Devices**<br>• **Innowalk**: A robotic walking device that supports upright, assisted walking.<br>• Clinical studies (in CP) found improvements in walking speed, endurance, and balance with robotic-assisted treadmill training (e.g., Lokomat).<br>**Augmented Reality Games**<br>• There is an AR-based active video game (ARRoW-CP) under study for gait training post-surgery in CP
**46-A3**	**What is the duration of botulinum toxin's effect in children with cerebral palsy?**	It depends a lot on the muscle injected, dose, and individual factors — but broadly:<br>• Clinical effects (reduced spasticity) typically last **3–6 months** in children with cerebral palsy.<br>• In one retrospective study of upper-limb injections, the mean duration was about **7.0 ± 3.0 months**.<br>• Some very-low-dose BoNT-A combined with therapy even gave effects lasting **10–12 months** in a few children.<br>• On the structural level, changes in muscle (like reduced muscle volume) can persist for **6 months** after injection and may not fully recover by that time.<br>So, while the "functional" spasticity-reducing effect is often in the 3–6 month range, there can be longer or shorter durations depending on many factors.
**47-A4**	**What can be done for my child with cerebral palsy who walks on their toes?**	Toe walking is very common in children with **cerebral palsy (CP)**, especially those with spasticity in the calf muscles. The management depends on the severity, the child's age, and whether it's flexible or fixed.<br>**1. Non-surgical / conservative options**<br>**a. Physical therapy**<br>• Stretching the calf muscles (gastrocnemius, soleus) regularly.<br>• Strengthening ankle dorsiflexors (muscles that lift the foot).<br>• Gait training and functional exercises.<br>• Balance and proprioception exercises.<br>**b. Orthoses**<br>• **Ankle-foot orthoses (AFOs):** Keeps the foot in a neutral position and prevents contractures.<br>• **Night splints:** Maintain stretch while sleeping to reduce calf tightness.<br>**c. Botulinum toxin (BoNT-A) injections**<br>• Temporarily reduce spasticity in calf muscles.<br>• Usually combined with stretching and orthoses.<br>• Effects last about **3–6 months**, sometimes longer.<br>**2. Medical / pharmacologic options**<br>• Rarely, oral medications like **baclofen** are used for generalized spasticity.<br>• Localized BoNT-A is preferred for focal spasticity like toe walking.<br>**3. Surgical options (for persistent or severe contractures)**<br>• **Tendon lengthening** (Achilles or gastrocnemius) if there's a fixed equinus deformity.<br>• Surgery is usually considered **after conservative measures fail** or if the child is older and deformity is fixed.

**48-A1**	**What are the different types of cerebral palsy?**	**1. Types Based on Motor Abnormality**<br>**a. Spastic CP (most common, ~70–80%)**<br>• **Cause:** Increased muscle tone (hypertonia) due to upper motor neuron damage.<br>• **Characteristics:** Stiff muscles, awkward movements, exaggerated reflexes.<br>• **Subtypes based on body distribution:**<br>○ **Spastic hemiplegia:** One side of the body (arm more affected than leg).<br>○ **Spastic diplegia:** Mainly legs; arms less affected.<br>○ **Spastic quadriplegia:** All four limbs affected; often severe.<br>○ **Spastic monoplegia:** One limb affected (rare).<br>• **Typical issues:** Toe walking, scissoring of legs, difficulty with fine motor skills.<br>**b. Dyskinetic / Athetoid CP**<br>• **Cause:** Basal ganglia involvement.<br>• **Characteristics:**<br>○ Slow, writhing, involuntary movements (athetosis).<br>○ Fluctuating muscle tone (sometimes floppy, sometimes stiff).<br>• **Subtypes:**<br>○ **Athetosis:** Slow, twisting movements.<br>○ **Choreoathetosis:** Combination of jerky (chorea) and writhing (athetosis) movements.<br>• **Typical issues:** Difficulty maintaining posture, speech problems.<br>**c. Ataxic CP**<br>• **Cause:** Cerebellar involvement.<br>• **Characteristics:**<br>○ Poor balance and coordination.<br>○ Tremors, unsteady gait (wide-based gait).<br>○ Difficulty with precise movements.<br>• **Typical issues:** Trouble with handwriting, fast movements, or walking on uneven surfaces.<br>**d. Mixed CP**<br>• **Cause:** Damage in multiple areas of the brain.<br>• **Characteristics:** Features of more than one type (commonly spastic + dyskinetic).<br>• **Typical issues:** Combination of stiffness, involuntary movements, and coordination problems.<br>**2. Classification by Body Parts (Distribution)**<br>• **Hemiplegia:** One side of the body.<br>• **Diplegia:** Legs more affected than arms.<br>• **Quadriplegia / Tetraplegia:** All four limbs involved.<br>• **Monoplegia:** Single limb involved.<br>• **Triplegia:** Three limbs affected (rare).
**49-A3**	**Can botulinum toxin injections and serial casting be used together in my child with cerebral palsy?**	**Botulinum toxin (Botox) injections and serial casting can be used together** in children with cerebral palsy (CP), and in fact, they are often **combined as part of a management strategy for spasticity**. Let me break it down carefully:<br>**1. Purpose of Each Treatment**<br>• **Botulinum toxin injections:**<br>○ Temporarily weaken overactive muscles (reduce spasticity).<br>○ Usually lasts 3–6 months.<br>○ Commonly injected into calf muscles (gastrocnemius, soleus) for ankle equinus, or other spastic muscles.<br>• **Serial casting:**<br>○ Gradually stretches spastic or contractured muscles and improves joint range of motion.<br>○ Typically done over 1–3 weeks, changing casts every few days.<br>○ Often applied to the ankle or wrist depending on which joints are affected.<br>**2. How They Work Together**<br>• Botulinum toxin **reduces spasticity**, making the muscles easier to stretch.<br>• Serial casting can then **maintain or improve the muscle length** and joint range of motion while the toxin is effective.<br>• Combining them often leads to **better functional gains** (e.g., improved gait, easier shoe fitting).<br>**3. Timing & Practical Considerations**

		• Botox is usually injected **first**, then serial casting is applied after a few days once spasticity starts to decrease.<br>• Casting is generally limited to a safe range of motion to avoid injury.<br>• Therapy and stretching are continued after cast removal to **maintain improvements**.<br>**4. Evidence**<br>• Multiple studies support that **botulinum toxin + serial casting** is more effective than either treatment alone for improving **ankle dorsiflexion and gait** in children with spastic CP.<br>• The combination is most commonly used for **lower limb spasticity**, especially **gastrocnemius/soleus muscles.**
**50-A3**	**What is the maximum number of times Botox can be injected into the same muscle in a child with cerebral palsy?**	There isn't a strict **absolute maximum number of times** botulinum toxin (Botox) can be injected into the same muscle in children with cerebral palsy, but there are **practical and safety guidelines** to follow:<br>**1. Typical Re-injection Interval**<br>• Botox effects usually last **3–6 months**.<br>• Re-injections are usually scheduled **no more frequently than every 3 months**.<br>• Shorter intervals increase the risk of **antibody formation**, which can reduce effectiveness over time.<br>**2. Cumulative Lifetime Considerations**<br>• Studies in children with CP have followed patients for **years**, with repeated injections to the same muscle every 3–6 months.<br>• There is **no defined upper limit**, but clinicians monitor for:<br>○ Muscle atrophy or weakness<br>○ Local side effects (pain, bruising, infection)<br>○ Development of neutralizing antibodies (rare)<br>• Most children tolerate **many cycles over several years**, especially when combined with therapy, casting, or orthoses.<br>**3. Clinical Guidelines**<br>• **American Academy of Neurology (AAN)** and **European consensus statements**:<br>○ Botox can be used **repeatedly for spastic muscles** as long as there is a good clinical response and no serious adverse effects.<br>○ Typically, injections are spaced **≥12 weeks apart**.<br>• **Max total dose** per session is calculated based on **weight and age**, not strictly the number of injections.
**51-A5**	**How can my child with cerebral palsy use their hands more effectively during daily activities?**	Improving hand function in a child with cerebral palsy (CP) focuses on **strength, coordination, and practical strategies** to make daily tasks easier. Here's a comprehensive guide:<br>**1. Occupational Therapy (OT)**<br>• **Assessment:** An OT can evaluate fine motor skills, hand strength, and grasp patterns.<br>• **Tailored exercises:** Activities are designed to improve:<br>○ **Grip strength** (e.g., squeezing therapy putty)<br>○ **Dexterity** (picking up small objects, stacking blocks)<br>○ **Bimanual coordination** (using both hands together for tasks like buttoning)<br>**2. Hand Positioning & Support**<br>• **Splints or orthoses:**<br>○ **Resting hand splints** can prevent contractures and maintain hand alignment.<br>○ **Functional splints** help the child hold objects more easily.<br>• **Adaptive equipment:**<br>○ Thick-handled utensils, Velcro fasteners, or modified pens can make self-feeding, dressing, or writing easier.<br>**3. Strengthening and Stretching**<br>• **Therapy putty, theraplast, or hand grips** can build strength gradually.<br>• **Stretching exercises** for tight fingers or wrists help maintain range of motion.<br>• **Weight-bearing activities on hands** (like crawling, push-ups against a wall) improve stability and control.<br>**4. Task-Specific Practice**<br>• **Daily activities as therapy:**<br>○ Encourage the child to **feed themselves**, **dress**, **brush teeth**, or **open containers** with supervision.

		○ Break tasks into **small, achievable steps**.<br>○ Repetition is key—**practicing functional tasks** helps the brain rewire for better control.<br>**5. Sensory and Motor Integration**<br>• Some children benefit from **sensory activities** that improve hand awareness, like:<br>○ Feeling different textures (sand, rice, water)<br>○ Finger painting<br>○ Playdough or clay molding<br>**6. Constraint-Induced Movement Therapy (CIMT)**<br>• The stronger hand is gently restrained for certain periods.<br>• Forces the **weaker or less-used hand to work**, improving function over weeks.<br>• Often guided by a therapist for **short, focused sessions**.
**52-A4**	**How does my child's motor function level influence their gait pattern?**	Your child's **motor function level** has a direct and significant impact on their **gait pattern**. To explain clearly, I'll break it down step by step, with a focus on common clinical frameworks like **Gross Motor Function Classification System (GMFCS)** for children with motor disorders, such as cerebral palsy, though similar principles apply more broadly.<br>**1. Gross Motor Function and Gait Ability**<br>Motor function level reflects the **child's ability to control muscles, coordinate movement, and bear weight**, all of which shape how they walk.<br>• **Higher motor function levels** (e.g., GMFCS I-II):<br>○ Can walk independently, often without aids.<br>○ Gait is close to typical but may show subtle deviations (e.g., toe-walking, mild crouch, or reduced step length).<br>○ They have more dynamic balance, allowing efficient weight transfer and smoother stride.<br>• **Moderate motor function levels** (e.g., GMFCS III):<br>○ Require assistive devices (walkers, crutches) for mobility.<br>○ Gait may be slower, with shorter step length, wider base of support, and increased energy expenditure.<br>○ Compensatory patterns may appear, such as hip hiking, circumduction, or vaulting, to clear the foot.<br>• **Lower motor function levels** (e.g., GMFCS IV-V):<br>○ Limited walking ability; may rely primarily on a wheelchair.<br>○ If gait occurs, it's often very slow, unstable, and requires maximal support.<br>○ Severe muscle weakness or spasticity leads to marked deviations (e.g., crouch, scissoring, equinus).
**53-A5**	**What strategies can help teach a new hand skill to my child with cerebral palsy?**	Teaching a new hand skill to a child with cerebral palsy (CP) requires a combination of patience, structured practice, and approaches tailored to their specific motor abilities.<br>**1. Break Skills into Steps**<br>• Teach one small movement at a time (e.g., open hand → grasp → lift → release).<br>**2. Use Repetition and Short Sessions**<br>• Practice 5–10 minutes several times a day.<br>• Repeat often, but avoid fatigue.<br>**3. Incorporate Play and Motivation**<br>• Turn tasks into games (stacking blocks, bubbles, putty).<br>• Praise effort and small successes.<br>**4. Adapt Objects and Positioning**<br>• Use larger or textured objects.<br>• Support the trunk and stabilize posture.<br>**5. Hand-Over-Hand Guidance**<br>• Guide movements initially, then gradually reduce help.<br>• Use both hands together when possible.<br>**6. Include Everyday Tasks**<br>• Practice skills during daily routines (feeding, dressing, drawing).<br>**7. Work With Professionals**<br>• Occupational and physical therapists can tailor exercises and provide adaptive tools.
**54-A3**	**What activities can enhance the therapeutic effects of**	For children with **cerebral palsy (CP)**, **botulinum toxin (BoNT-A) injections** help reduce spasticity temporarily, but they are **most effective when combined with targeted therapies**. The injections alone won't improve strength, motor control, or function — they

	**botulinum toxin in my child with cerebral palsy?**	simply make muscles easier to stretch and use. Here's a detailed breakdown of activities and interventions that can enhance the therapeutic effects:<br>**1. Physical Therapy**<br>• **Goal:** Improve strength, flexibility, and motor control while the muscle is relaxed.<br>• **Examples:**<br>○ Stretching of spastic muscles (to maintain length gained after injection)<br>○ Strengthening of antagonistic muscles (opposite to the injected muscles)<br>○ Task-specific training, e.g., walking, reaching, or grasping<br>• **Frequency:** Often several sessions per week for optimal benefit<br>**2. Occupational Therapy**<br>• **Goal:** Improve functional use of the upper and lower limbs in daily activities.<br>• **Examples:**<br>○ Fine motor skill training (picking up small objects, buttoning)<br>○ Hand positioning and grasp patterns<br>○ Adaptive equipment training if needed<br>**3. Serial Casting / Splinting**<br>• **Goal:** Maintain or increase muscle length and prevent contractures after injections.<br>• **Types:**<br>○ **Serial casting:** Plaster or fiberglass casts that gradually stretch the muscle<br>○ **Orthoses / splints:** Night or day use to maintain range of motion<br>**4. Constraint-Induced Movement Therapy (CIMT)**<br>• **Goal:** Encourage use of a weaker or affected limb by restraining the stronger one.<br>• **Effect:** Can improve motor control and functional use, particularly after BoNT-A in upper limb muscles.<br>**5. Gait Training**<br>• **Goal:** Improve walking mechanics and functional mobility.<br>• **Examples:**<br>○ Treadmill training<br>○ Balance
**55-A4**	**Are orthotic devices necessary to support walking in my child with cerebral palsy?**	The answer depends on your child's specific needs, the type and severity of cerebral palsy (CP), and their functional abilities.<br>**1. Purpose of Orthotic Devices**<br>Orthotic devices (like ankle-foot orthoses, or AFOs) are used in children with CP to:<br>• **Improve stability**: They help maintain proper alignment of the feet and ankles, reducing falls.<br>• **Enhance walking efficiency**: Proper support can make walking less tiring.<br>• **Prevent deformities**: CP can lead to muscle contractures or abnormal bone development; orthotics can slow or prevent these.<br>• **Support muscle function**: They can help muscles work more effectively by positioning joints optimally.<br>**2. Factors Determining Necessity**<br>Not all children with CP need orthotics. Key considerations include:<br>• **Type and severity of CP**: Children with spastic diplegia or hemiplegia are more likely to benefit from AFOs.<br>• **Walking ability**: Children who can walk but have toe-walking, crouch gait, or frequent falls often benefit from orthotics.<br>• **Muscle tone and strength**: High spasticity or weak muscles may require support.<br>• **Growth and joint alignment**: Orthotics may be prescribed to prevent progressive deformities.
**56-A3**	**Does Botox cause any harm to the muscles in children with cerebral palsy?**	Botulinum toxin (Botox) is commonly used in children with cerebral palsy (CP) to reduce spasticity (muscle overactivity). Understanding whether it causes harm requires a careful look at both **short-term and long-term effects**.<br>**Potential Harm or Side Effects**<br>Most children tolerate Botox injections well, but there are some possible concerns:<br>**Common, usually mild effects:**<br>• Soreness or swelling at the injection site<br>• Temporary weakness in the injected muscle

		• Fatigue or flu-like symptoms<br>**Less common, more serious effects:**<br>• Spread of toxin to other muscles causing generalized weakness (rare)<br>• Difficulty swallowing or breathing if injected near the neck or throat (very rare)<br>• Allergic reactions (extremely rare)<br>**Muscle-specific concerns:**<br>• Some studies suggest **repeated Botox injections may slightly reduce muscle size or strength**, especially if injections are frequent over years.<br>• However, most research shows that **the benefits in reducing spasticity and improving function outweigh these risks** when injections are appropriately spaced and combined with physical therapy.
**A1: Information about cerebral palsy, A2: Exercise and Physical Activity, A3: Botulinum Toxin / Serial Casting / Muscle Lengthening, A4: Gait and Balance, A5: Hand Function.**		
